# Assessing Genetic Structure in Common but Ecologically Distinct Carnivores: The Stone Marten and Red Fox

**DOI:** 10.1371/journal.pone.0145165

**Published:** 2016-01-04

**Authors:** Mafalda P. Basto, Margarida Santos-Reis, Luciana Simões, Clara Grilo, Luís Cardoso, Helder Cortes, Michael W. Bruford, Carlos Fernandes

**Affiliations:** 1 Ce3C – Centre for Ecology, Evolution and Environmental Changes, Faculdade de Ciências, Universidade de Lisboa, Lisboa, Portugal; 2 Cardiff School of Biosciences, Cardiff University, Cardiff, United Kingdom; 3 Centro Brasileiro de Estudos em Ecologia de Estradas/Programa de Pós-graduação em Ecologia Aplicada, Universidade Federal de Lavras, Lavras, Minas Gerais, Brasil; 4 Departamento de Ciências Veterinárias, Escola de Ciências Agrárias e Veterinárias, Universidade de Trás-os-Montes e Alto Douro (UTAD), Vila Real, Portugal; 5 Laboratório de Parasitologia Victor Caeiro, Instituto de Ciências Agrárias e Ambientais Mediterrânicas (ICAAM), Universidade de Évora, Évora, Portugal; University of Regina, CANADA

## Abstract

The identification of populations and spatial genetic patterns is important for ecological and conservation research, and spatially explicit individual-based methods have been recognised as powerful tools in this context. Mammalian carnivores are intrinsically vulnerable to habitat fragmentation but not much is known about the genetic consequences of fragmentation in common species. Stone martens (*Martes foina*) and red foxes (*Vulpes vulpes*) share a widespread Palearctic distribution and are considered habitat generalists, but in the Iberian Peninsula stone martens tend to occur in higher quality habitats. We compared their genetic structure in Portugal to see if they are consistent with their differences in ecological plasticity, and also to illustrate an approach to explicitly delineate the spatial boundaries of consistently identified genetic units. We analysed microsatellite data using spatial Bayesian clustering methods (implemented in the software BAPS, GENELAND and TESS), a progressive partitioning approach and a multivariate technique (Spatial Principal Components Analysis-sPCA). Three consensus Bayesian clusters were identified for the stone marten. No consensus was achieved for the red fox, but one cluster was the most probable clustering solution. Progressive partitioning and sPCA suggested additional clusters in the stone marten but they were not consistent among methods and were geographically incoherent. The contrasting results between the two species are consistent with the literature reporting stricter ecological requirements of the stone marten in the Iberian Peninsula. The observed genetic structure in the stone marten may have been influenced by landscape features, particularly rivers, and fragmentation. We suggest that an approach based on a consensus clustering solution of multiple different algorithms may provide an objective and effective means to delineate potential boundaries of inferred subpopulations. sPCA and progressive partitioning offer further verification of possible population structure and may be useful for revealing cryptic spatial genetic patterns worth further investigation.

## Introduction

In conservation biology, interest is growing on the population genetic status and viability of species traditionally classed as ‘common’ [1,2,3] since many abundant species are under the impact of human population growth and actions [4]. In the face of the current biodiversity crisis, sound management and conservation requires genetic information to preserve species as dynamic entities capable of coping with environmental change [5]. In this context, the accurate identification of population genetic structure is crucial [6,7]. Moreover, detailed genetic sampling of threatened species, especially across broad geographic areas, may prove difficult due to their generally low abundance or local extinction. Thus, a deeper understanding of the genetic patterns of abundant species may be useful to unravel contemporary and historical factors also influencing rare and threatened species [1,8].

Much remains unknown about the genetic consequences of habitat fragmentation and heterogeneity on common, vagile and apparently continuously distributed species. These species are generally assumed to have absent or negligible population structure or an isolation-by-distance pattern (IBD). However, an increasing number of studies are showing that unexpected patterns of genetic differentiation can be observed in mobile and widespread habitat generalists [9,10,11,12]. These patterns may be complex and difficult to interpret, among other reasons because they may have been influenced by historical events, topographic features, recent landscape fragmentation, and/or habitat preferences [9,13,14,15]. Past landscape structure and long-standing barriers to dispersal such as rivers [16,17] can leave a lasting legacy on spatial genetic patterns of modern populations, particularly when habitat fragmentation and ecological or behavioural processes restrict current gene flow [18]. Alternatively, higher migration rates may erode signatures of previous genetic differentiation [19] and prevent structuring and divergence across semi permeable barriers [20].

Microsatellites [21] provide efficient and cost-effective markers to address many questions in molecular ecology [22] and have been shown to be particularly powerful in studies assessing genetic structure in widespread and mobile species (e.g. [9,11,23]). Inference of population structure has benefited from increasingly sophisticated and sensitive individual-based statistical methods [24,25,26]. Among these, most have been developed within a Bayesian framework and can use information on the geographic coordinates of individuals [27,28,29]. It has also been shown that applying a hierarchical analysis of population clustering can help identifying partitions with low levels of genetic differentiation (e.g. [30]). Lately, Bayesian clustering methods have been criticised for being model-based, for relying on assumptions that can limit their applicability and may be difficult to verify [26], for being unable to estimate the magnitude of spatial correlations in the presence of isolation-by-distance [25], and for being sensitive to weaknesses in the sampling [31,32]. It has therefore been suggested that a spatial ordination method may be useful to explore the fraction of the genetic variability that is spatially structured [33]. Spatial Principal Components Analysis (sPCA, [25]), an adaptation of PCA that optimises the variance of the principal components and their spatial autocorrelation, can be an efficient method to recognise different types of spatial structure that may occur in genetic data [33]. In this study, we wanted to explore the usefulness of a particular combination of Bayesian and multivariate techniques to draw robust inferences about population structure and spatial genetic patterns [30,34,35].

Studies on large carnivores (e.g. [10,11,13]) have provided important insights into how habitat barriers, habitat transitions, natal habitat-biased dispersal, and ecological preferences can generate and maintain population structure. However, similar investigations on mesocarnivores are comparatively rare [12,14].

Here we compared the genetic structure of two mesocarnivores across the fragmented and heterogeneous landscape of Portugal. We focused on the stone marten, *Martes foina*, which in Portugal seems to be forest-adapted [36] and sensitive to forest fragmentation [37], and the red fox, *Vulpes vulpes*, an opportunistic generalist that is highly plastic and resilient to anthropogenic landscape change [38,39]. The stone marten has a widespread distribution throughout the Palearctic region [40] and when in sympatry with the pine marten (*Martes martes*), has been described as a habitat generalist with synantropic behaviour [41], whereas in areas in which the pine marten is absent, as in most of the Iberian Peninsula, stone martens occur in higher quality habitats (forested and mosaic habitats—[36,42]). Despite the many previous population genetic studies on species within the genus *Martes* using microsatellites (e.g. [43,44]), very few have been carried out on stone martens [45,46,47]. The red fox is the most widespread terrestrial wild carnivore in the world, with a continuous Holarctic distribution ranging from the arctic tundra to temperate deserts [40], and consequently is one of the best studied carnivore species (reviewed in [38]). However, population genetic studies in the red fox using microsatellites are still relatively limited [14,19,48,49,50], and completely lacking in southern Europe.

We wanted to test the hypothesis that the stone marten, a common and generalist carnivore but with stricter ecological requirements in Iberia, may exhibit population genetic structure, whereas the red fox, a species with a greater ecological plasticity, may not. For each of the two species we (i) tested for the presence of distinct genetic clusters using spatial Bayesian clustering methods (implemented in the software BAPS [29], GENELAND [27] and TESS [28]), a progressive partitioning approach [30], and a multivariate technique (sPCA, [25]); (ii) assessed genetic diversity and isolation-by-distance patterns in the whole data set and within inferred genetic units; and (iii) estimated the level of differentiation and ongoing gene flow among genetic units. We discuss and compare the results for the two species and relate them to our hypothesis. Population genetic surveys of wild mammals across Portugal are very rare [51,52], and this study also aims to help to fill this gap.

## Materials and Methods

### Ethics statement

Hair and tissue samples were obtained from road-kills, live-trapped animals subsequently released at the point of capture (stone martens), and legally hunted animals (red foxes) by hunters and hunting associations. Hunted animals were shot during the hunting season, under the rules of the Portuguese hunting law. No animals were killed specifically for this study. No Government approval or licenses were required for sampling road-kills or legally hunted animals. Permissions for trapping and sampling live animals were obtained from the Instituto da Conservação da Natureza e das Florestas (ICNF) (240/2011/CAPT; 241/2011/CAPT; 254/2009/CAPT).

### Sampling and Microsatellite Genotyping

**Fig 1:**
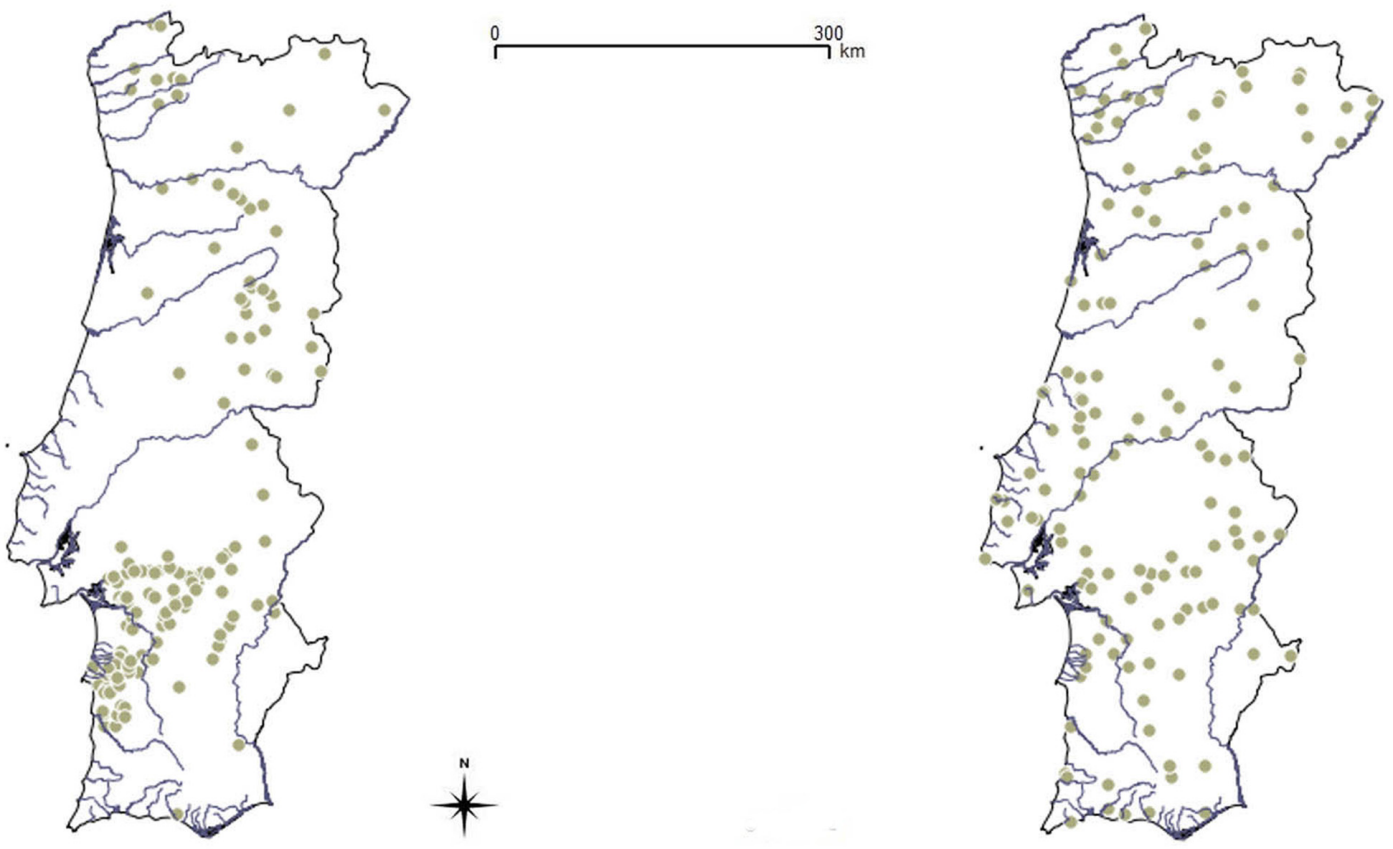
Geographic location of samples in Portugal. Geographic location of samples of stone martens (left map) and red foxes (right map). Lines represent country borders and main watercourses.

Samples (n = 159 for stone marten; n = 143 for red fox) were collected between 2002 and 2011. All samples were geo-referenced (Tables A and B in S1 File). For red foxes our sampling encompassed the entire country, while for stone martens there was a gap in the west-central region of the country where no samples were obtained despite exhaustive surveys (Fig 1).

Tissue samples were preserved in a salt-saturated solution of 20% DMSO in water or in absolute ethanol and stored at -20°C and hair samples were kept at room temperature. DNA was extracted using the DNeasy Tissue kit (QIAGEN, Hilden, Germany). To monitor potential contamination, we included a negative extraction control in each extraction session. Since the pine marten is also present in the north of Portugal, species identification of the marten samples from this area was ascertained using species-specific mitochondrial DNA markers [53].

Stone marten samples were genotyped for 12 microsatellite loci, with species-specific primers described in [54] (Mf 1.1, Mf 1.11, Mf 1.3, Mf 2.13, Mf 3.2, Mf 3.7, Mf 4.10, Mf 4.17, Mf 6.5, Mf 8.7, Mf 8.8 and Mf 8.10) (Table A in S2 File). Red fox samples were genotyped for 10 microsatellite loci, using domestic dog primers known to work in foxes [55] (FH2174, FH2189, FH2261, FH2302, FH2318, FH2412, FH2541, FH2613, FH3320, PEZ16) (Table B in S2 File). Microsatellites were amplified via polymerase chain reaction (PCR) in a GeneAmp 9700 Thermal Cycler (Applied Biosystems, Warrington, UK) and in a total volume of 10 μl: 2μl of DNA extract, 1X PCR Buffer, 3 mM MgCl_2_, 0.2 mM of each dNTP (Bioline, London, UK), 0.5 μM of each primer plus 0.5 μM of labelled M13 tag oligonucleotide [56], 0.5 μg/μl bovine serum albumin (BSA; New England Biolabs, Herts, UK), and 0.5U of HotSurf Taq DNA Polymerase (Stabvida, Lisbon, Portugal). The loci were amplified in singleplex reactions. Thermal cycling was performed with the following general protocol: initial denaturation at 94°C for 15min, followed by five cycles at 94°C for 30s, the reverse primer’s annealing temperature (Ta) + 10°C for 30s and 72°C for 30s, followed by 10 cycles at 94°C for 30s, Ta + 5°C for 30s and 72°C for 30s, and finally 22 cycles at 94°C for 30s, Ta for 30s and 72°C for 30s. Final extension was at 72°C for 20min. Contamination was monitored using extraction and PCR negative controls. Fragment lengths of PCR products were determined with an ABI PRISM 3130 Genetic Analyser (Applied Biosystems, Warrington, UK) using GeneScan-500 ROX size standard, and analysed with GeneMapper 3.2 (Applied Biosystems, Foster City, USA). Genotyping was validated by re-amplification and re-analysis of 25% of the samples for each locus. The error rate per reaction, calculated as the number of incorrect genotypes divided by the total number of reactions used for comparison, was 0.009 in the stone marten data set and 0.008 in the red fox data set.

### Population Structure

#### Bayesian clustering and progressive partitioning

Multilocus genotypes for stone martens and red foxes were analysed using spatial Bayesian clustering methods implemented in BAPS v.5 [29], GENELAND v.4.0.3 [27] and TESS v.2.3.1 [28] to determine the most likely number of clusters (K). For comparison, we also used one of the most popular aspatial Bayesian clustering algorithms, the model with admixture and correlated allele frequencies available in STRUCTURE [57,58]. Whenever feasible the same parameters were chosen to make the results as comparable as possible. Parameters and models are summarised in Table 1. In BAPS, K was determined by evaluating the 10 best partitions, in terms of marginal likelihood and posterior probability for the number of clusters, across runs with different values of maximum K (10 replicates for each value). In GENELAND we conducted analyses using both the uncorrelated and correlated allele frequency models. The correlated frequency model may be more powerful at detecting subtle differentiation, but it may also be more sensitive to departures from model assumptions (e.g. presence of isolation-by-distance) and more prone to algorithm instabilities than the uncorrelated frequency model [59,60]. The choice of K was based on the histogram of estimated K for each run, the highest mean posterior density across replicates, and in a detailed probability map of assignments to evaluate the degree of uncertainty of the estimated cluster memberships. In TESS we used both the no-admixture [28,61] and the admixture [62] models. Admixture models are more efficient in the presence of clines in allele frequencies and admixture proportions resulting from fusion events [62,63], but the TESS model without admixture appears to be more robust to IBD [64] and may provide an upper bound on the number of clusters in the data [65]. In the analyses assuming admixture, we tested both the CAR and the BYM models with linear trend surfaces [62] to define the spatial prior for admixture proportions. To decide which K_max_ (and K) may provide the best fit to the data, we plotted the deviance information criterion (DIC) against K_max_ and considered the values for which the DIC first reached a plateau [62]. Because this approach sometimes selects models in which K_max_ is greater than the effective number of clusters K [62], we also examined when bar plots of estimated membership probabilities stabilized (i.e. when no additional clusters are detected at higher values of K_max_) and the log-likelihood values. Finally, in STRUCTURE v.2.3.4 we ran 10 replicate runs for each potential number of genetic clusters, and the results were then used in STRUCTURE HARVESTER v.0.9.94 [66] to select the K value associated with the highest mean posterior probability of the data (Ln P(D)).

**Table 1:** Summary of the Bayesian clustering analysis of population structure and respective estimates of the number of genetic clusters.

Software	Models	Tested values of K	Inferred *K*	q-value threshold for
			(stone marten / red fox)	cluster membership
**BAPS**	Spatial clustering of individuals	K = 5,10,15 (10 runs for each K)	3 / 1	0.9
**TESS**	No-admixture (ψ = 0.6)		3 / 1	0.9
	CAR admixture model (updating ψ and variance)		3 / 2	0.5
	BYM admixture model (updating ψ and variance)		3 / 2	0.5
	50,000 sweeps (after a burn-in of 10,000)	K = 2–11 (10 runs for each K)		
**GENELAND**	Uncorrelated frequency		3 / 2	0.9
	Correlated frequency		6 / 2	None
	Spatial model with coordinate uncertainty of 1,000 m	K = 1–11 (10 runs for each K)		
	Maximum rate of Poisson process = number of samples			
	Maximum number of nuclei = three times the sample size			
	To infer K: 500,000 iterations; at the inferred K: 200,000 iterations			
	Thinning = 1,000; burn-in of 1,000 in the MCMC post-processing			
**STRUCTURE**	Admixture and correlated allele frequencies	K = 1–11 (10 runs for each K)	3 / 1	0.5
	1,000,000 iterations (after a burn-in of 100,000)			

The clustering solution identified by each method was mapped using Quantum GIS v.1.8.0 (Quantum GIS Development Team, 2011) to plot the cluster membership for each individual from the best run for the most likely value of K.

To assess the presence of additional genetic structure we used a progressive partitioning approach forcing K = 2 within identified clusters [30]. Individuals were only assigned to a new partition when they had a membership probability higher than 0.5. The procedure was repeated until the entire set of individuals within a cluster remained assigned to a single cluster or the majority of the individuals had approximately 50% assignment to each of the two clusters.

#### Multivariate analysis

sPCA was performed in R v.3.0.0 (R Development Core Team, 2013) using the packages adegenet [67], ade4 [68] and spdep [69]. The analysis consisted of a centred, scaled PCA using Moran’s I test to detect spatial structuring in the PCA scores and cluster individual genotypes, and the data were subsequently analysed by sPCA, in which the connection network was defined using the Delaunay triangulation (type = 1) and the neighbourhood was based on pairwise geographic distances (type = 5). In the connection network for, respectively, stone martens and red foxes, individuals separated by less than 65 km or less than 50 km (which resulted in all samples having at least one connection) were considered neighbours. Two Monte Carlo tests were performed to assess the significance of global and local structures (with 9,999 permutations). Global scores can identify genetically distinguishable groups, clines and intermediate individuals, whereas local scores may reflect differentiation between neighbouring individuals.

### Genetic Variation

Genetic variation was quantified using standard summary statistics. We estimated the number of alleles per locus (N_A_), observed (H_O_) and unbiased expected (UH_E_) heterozygosities using GenAlEx 6.5 [70]. Hardy–Weinberg equilibrium (HWE) and linkage equilibrium (LE) of the microsatellites were tested in GENEPOP v.4.2 [71]. A sequential Bonferroni correction for multiple tests was used to adjust significance levels [72]. The inbreeding coefficient (F_IS_) per locus was estimated using GENETIX v.4.05.2 [73] and their significance was assessed by 1,000 permutations of alleles among individuals. To check for the presence of null alleles under the assumption of HWE we used MICRO-CHECKER v.2.2.3 [74] and a significance level of 99%. For the inferred genetic units, the average allelic richness (AR) and private allelic richness (pAR) over loci were calculated in HP-RARE v.1.0 [75], using a rarefaction procedure to account for unequal sample sizes [76]. For the pAR estimates we considered a minimum allele frequency (MAF) of 0.01 to account for sampling errors.

While most red fox samples were collected between 2008 and 2011, stone marten samples were gathered between 2002 and 2011 and thus tested for temporal genetic homogeneity. For this purpose, we used a permutation test in FSTAT v.2.9.3.2 [77] to compare estimates of A_R_, H_O_, H_E_, and F_ST_ from samples collected before and after 2008. Samples were grouped before and after 2008 to obtain similar-sized data sets.

The software POWSIM v.4.1 [78] was used to evaluate the statistical power of our microsatellite panels to detect genetic differentiation. Simulations were run with various combinations of N_e_ (effective population size) and t (generations of drift before sampling) to yield F_ST_ values of 0.01 and 0.05. One thousand simulated data sets were generated for each scenario and the proportion of significant outcomes (P < 0.05), an estimate of power, was determined using Fisher’s method to combine exact P-values across loci.

Population structure and spatial genetic patterns may be due to factors such as bottlenecks or relatedness [17,79,80]. Increased genetic drift between bottlenecked populations may result in genetic structure [51,79]. Each inferred subpopulation was tested for evidence of recent genetic bottlenecks using the software BOTTLENECK v.1.2.02 [81]. Three models of microsatellite mutation were considered: the infinite alleles model (IAM), the stepwise mutation model (SMM) and the two-phase model (TPM), the latter weighted to 95% and 78% SMM with a variance for mutation size set to 12, following recommendations by [81] and [82], respectively. Significance of heterozygosity excess over all loci, indicative of a recent bottleneck, was assessed with sign and Wilcoxon signed rank tests. We also analysed the distribution of allele frequencies, which is expected to be L-shaped under mutation-drift equilibrium and to exhibit a characteristic ‘mode shift’ in bottlenecked populations [83].

Relatedness between individuals was estimated using COANCESTRY v.1.0.1.2 [84]. The best likelihood estimator was determined by Monte Carlo simulations of 1,000 dyads for four relationships categories (parent-offspring, full-siblings, half-siblings and unrelated). The relatedness (*r*) for each dyad was calculated using all the estimators available in COANCESTRY (see [84]) and compared with the true simulated relatedness values (based in the observed allele frequencies). The estimator that best balanced between highest correlation values and lowest variance was chosen for subsequent analyses. In these analyses, we estimated and compared the mean pairwise relatedness overall and among inferred clusters. Specifically, we wanted to assess whether relatedness was low and evenly distributed across the study area.

### Isolation-by-distance

Bayesian clustering methods can overestimate genetic structure in individual-based data sets characterized by isolation-by-distance [31]. Presence of IBD was tested, both in the whole data set and within inferred subpopulations, by analysing the genetic distance between pairs of individuals as a function of geographic distance in the program GenAlEx. Pairwise genetic distance was estimated using Rousset’s distance [85] and Nason’s kinship estimator [86] in SPAGEDI v.1.4 [87].

### Genetic Differentiation

The level of genetic differentiation was estimated by pairwise F_ST_ [88] using GENETIX and significance was assessed by 1,000 permutations. G”_ST_ [89] and Jost’s D_EST_ [90], which correct the dependency of F_ST_ for the amount of within-population variation, were also calculated using GENODIVE v.2.0b24 [91].

### Recent Migration

Recent bidirectional migration rates were estimated using the program BAYESASS v.3.0 [92]. Ten independent runs of the algorithm were performed, each with 10^7^ iterations after a burn-in of 10^6^ iterations. The program TRACER v.1.5 [93] was used to check for convergence and estimate effective sample sizes (ESS), mean values, and 95% highest posterior density (HPD) intervals of the migration rate parameters in the combined runs. To identify possible first-generation migrants we used the software GENECLASS2 [94]. The Bayesian criterion of Rannala and Mountain [95] was employed to estimate the likelihood of each individual genotype within the population where it has been sampled. Probability values were calculated using the Monte Carlo resampling method of Paetkau et al. [96], with 1,000 simulated individuals and an alpha level of 0.01.

## Results

### Population Structure

**Fig 2:**
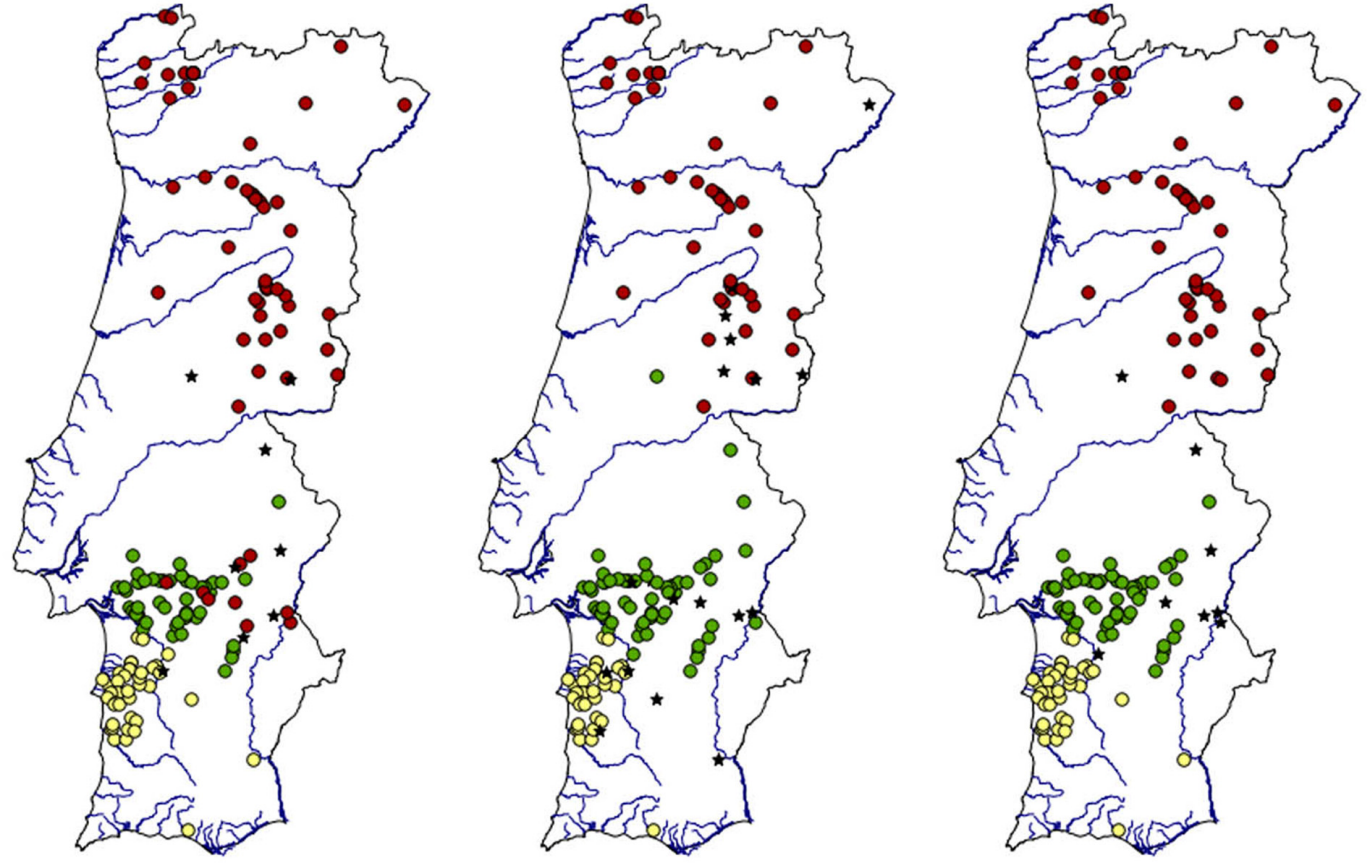
Cluster membership of stone marten individuals. Cluster membership of stone marten individuals with a membership probability ≥ 0.9 in the best run of, respectively from left to right, BAPS (K = 3), TESS with no admixture (K = 3) and GENELAND for the uncorrelated frequency model (K = 3). Stars represent non-assigned individuals (i.e., with membership coefficients < 0.9). Lines represent country borders and main watercourses.

In the stone marten, all Bayesian clustering methods yielded the same optimal number of clusters (K = 3) (Fig 2; Fig A in S1 File), with the exception of the correlated frequency model in GENELAND (K = 6, Fig A in S1 File). The latter, like the others, also identified a cluster in the southwest (yellow) and another cluster in the south (green), but suggested an additional small cluster in the south and subdivided the samples from the north into three clusters along a latitudinal axis (Fig A in S1 File). However, unlike all other models, the individual probabilities of cluster membership obtained with the correlated frequency model in GENELAND were very low (< 0.5). Moreover, the analysis using the correlated frequency model in GENELAND with varying K inferred seven clusters (K = 7), while the subsequent runs with K fixed to seven indicated six clusters (K = 6). The presence of ‘ghost’ clusters [97] can be seen as an indication of departure of the data from modeling assumptions [98].

The different clustering algorithms supporting K = 3 suggested a broadly similar spatial distribution of the identified clusters, but with some discrepancies in the cluster membership of individuals. Notably, in BAPS and STRUCTURE some individuals in the south clustered with the individuals from the north, and the CAR admixture model in TESS grouped several individuals in the southwest with those from the south. STRUCTURE also grouped a few individuals in the north and south with those from the southwest, thus giving the less geographically consistent clustering solution among all methods tested. Conversely, the uncorrelated frequency model in GENELAND and both the no-admixture and the BYM admixture models in TESS produced very similar clustering results (Fig 2; Fig A in S1 File).

In BAPS, the no-admixture model in TESS and the uncorrelated frequency model in GENELAND, the percentage of individuals with membership coefficients ≥ 0.9 were 92%, 88% and 95%. The percentage of individuals assigned with < 0.8 probability was respectively 3%, 6% and 3%, and only in BAPS there was one individual with an assignment probability < 0.5. The proportions of misassignments (with *q*-values ranging from 0.47 to 0.84 in BAPS, from 0.5 to 1.0 in TESS, and from 0.5 to 0.97 in GENELAND) were respectively 7%, 6% and 3% (Fig 2). In the analyses with admixture in TESS (CAR and BYM) and STRUCTURE, the percentage of individuals with < 0.7 probability was 35%, 28% and 38%, but the percentage of individuals with ancestry proportion < 0.5 was low at 0%, 4% and 8%, respectively. Also respectively, only 12%, 5% and 15% of the individuals were not assigned to the population in which they were sampled (Fig A in S1 File). The value of the admixture parameter (α) in STRUCTURE was 0.21, implying that most individuals are essentially from one cluster or another [99]. In BAPS, the admixture analysis confirmed the individual assignments from the mixture analysis and no admixed individuals were detected (α = 0.05).

No-admixture models may be useful to identify the maximal number of clusters in the data [65,100,101] and may be more powerful than admixture models at detecting subtle structure [99]. Given this and the agreement between both types of methods on the number of genetic clusters, we focused on the clustering solutions given by the no-admixture algorithms (Fig 2). Specifically, a consensus matrix was constructed containing only individuals assigned to the same cluster by BAPS, the no-admixture model in TESS, and the uncorrelated frequency model in GENELAND (126 out of 159, i.e. 79%). A membership threshold of 0.9 was used to assign each individual to a cluster when building the consensus clustering solution. A threshold of 0.9 seemed a reasonable conservative choice, so that only individuals with very high cluster membership coefficients were assigned [102,103], while higher thresholds were deemed unnecessary because the final clustering solution was based on a strict consensus of the results of the individual algorithms.

**Fig 3:**
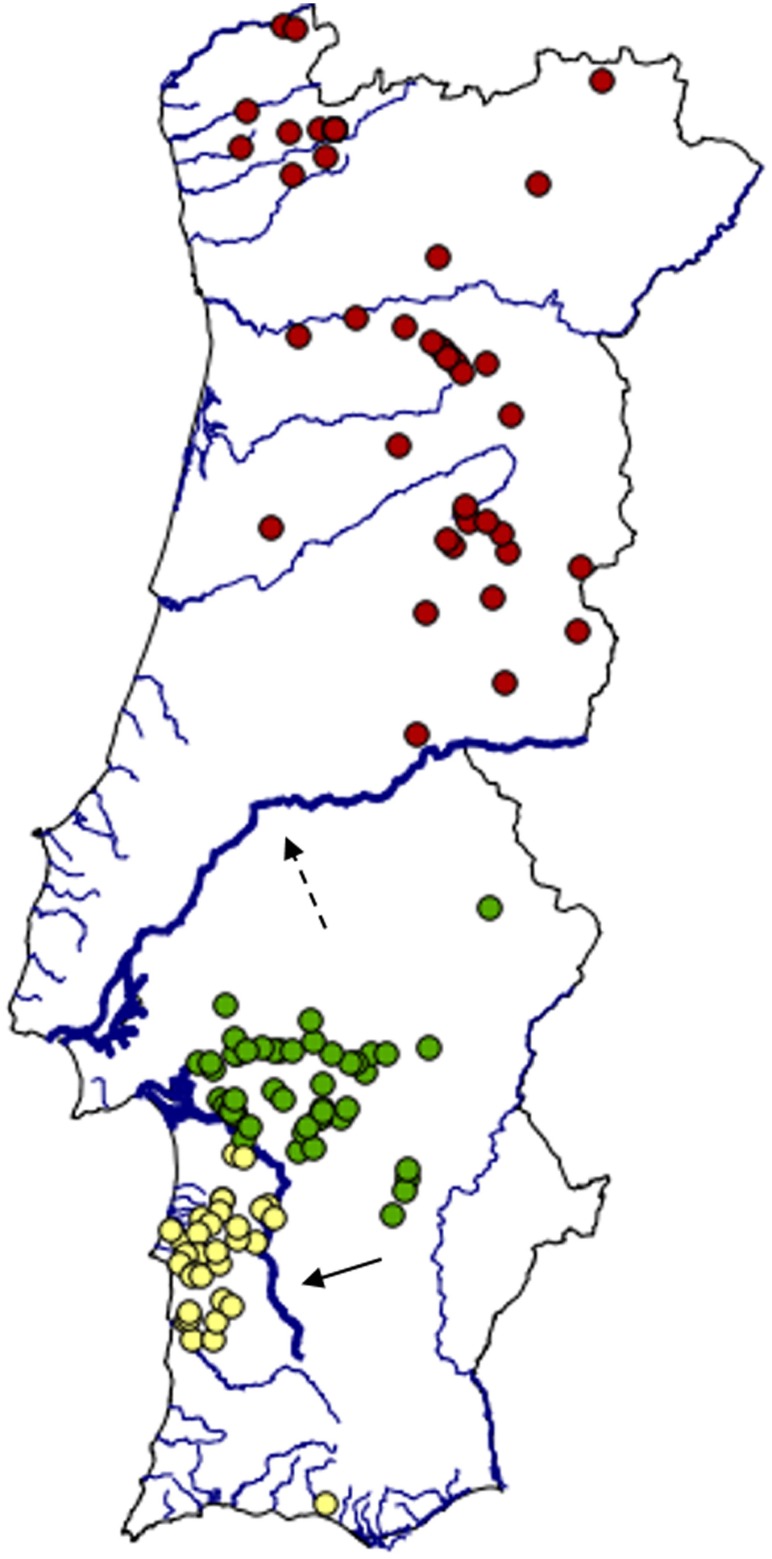
Bayesian consensus for the population structure of stone martens in Portugal. Lines represent country borders and main watercourses. The rivers Tagus and Sado are highlighted using thicker lines and indicated by the dashed and solid arrows, respectively.

Although there was broad agreement between the clustering obtained with different no-admixture models, they also showed some differences in terms of individual cluster membership (Fig 2). The consensus clustering solution, however, clarified the genetic structure and allowed a more explicit delineation of the potential boundaries of the inferred genetic units (Fig 3). Given their geographic locations in Portugal, the identified subpopulations are hereafter designated as ‘North’ (red dots), ‘South’ (green dots) and ‘Southwest’ (yellow dots).

Progressive partitioning was used to exhaustively test for further sub-structuring within the clusters obtained with the no-admixture models. No further subdivision was detected, except for the northern cluster in the GENELAND analysis (Fig B in S1 File).

**Fig 4:**
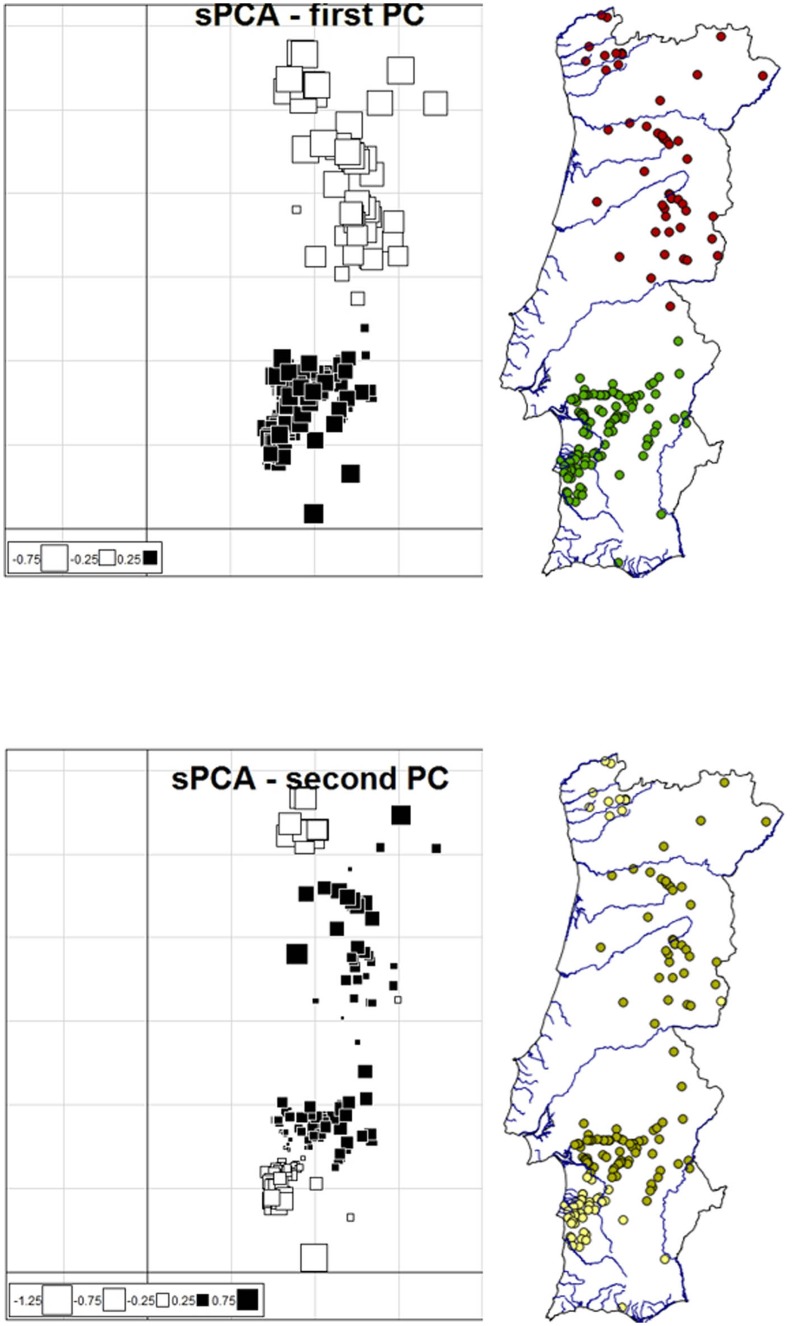
Genetic structure of stone martens in Portugal as assessed by sPCA. Shown are the first Principal Component (PC) and second PC, and the respective mappings of cluster membership. Lines in both maps represent country borders and main watercourses.

In the sPCA, two global patterns were identified in the eigenvalues barplot and the decomposition of each eigenvalue into its spatial autocorrelation and variance components (Fig C in S1 File). The first global component showed a north-south differentiation and the second global component showed a split in the south (Fig 4), both results being consistent with those obtained in the Bayesian analyses. The second global component also indicated a subdivision in the north partly similar to that found by the progressive partitioning analysis in GENELAND.

**Fig 5:**
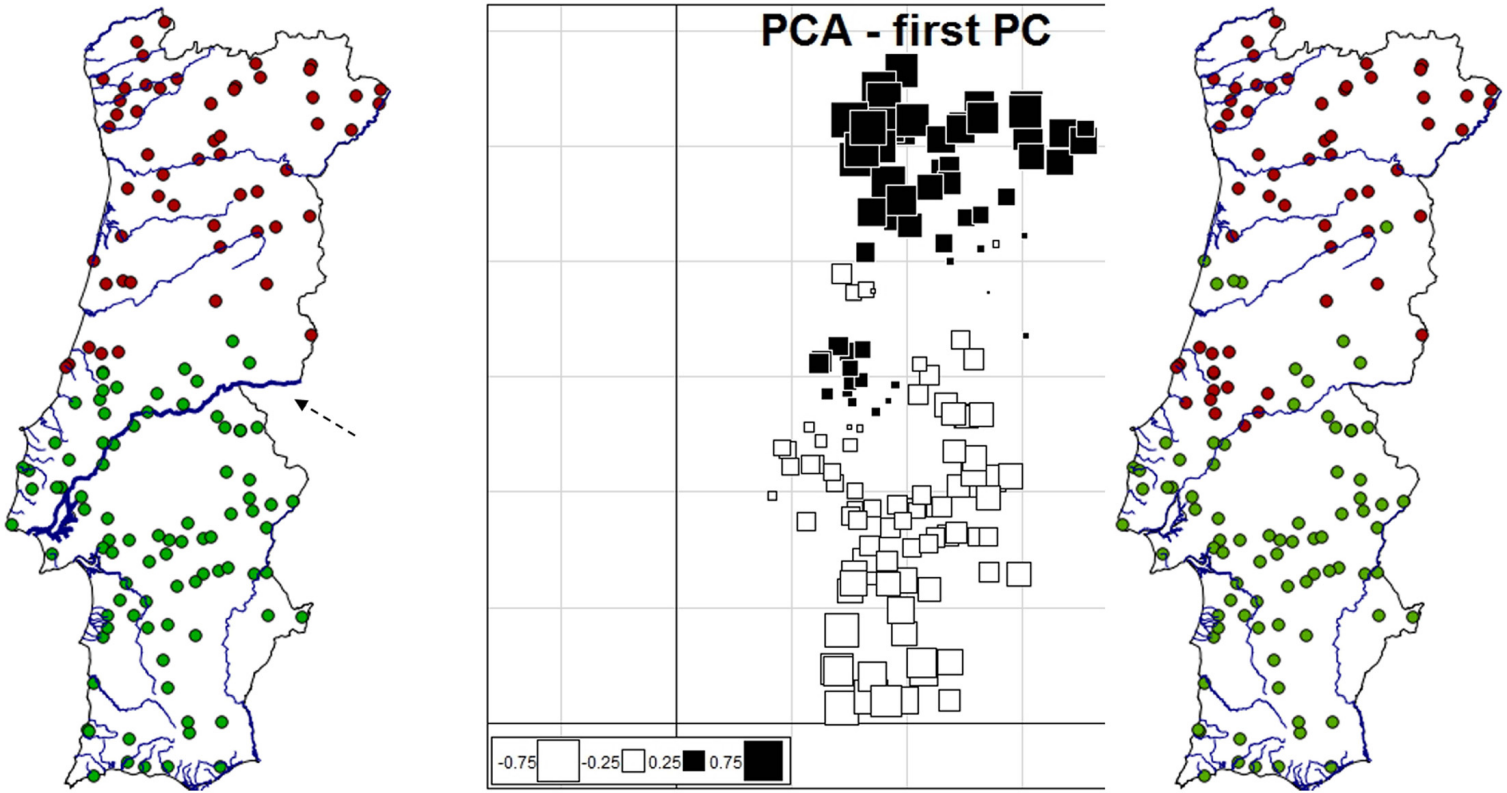
Cluster membership and genetic structure of red fox individuals in Portugal as inferred by the uncorrelated frequency model in GENELAND and sPCA. On the left, cluster membership of red fox individuals with a membership probability ≥ 0.9 by the uncorrelated frequency model in GENELAND (the River Tagus is indicated by a dashed arrow); On the middle and right, genetic structure as assessed by sPCA; shown are the first Principal Component (PC) and the respective mapping of cluster membership. Lines in both maps represent country borders and main watercourses.

Regarding the red fox, the different Bayesian clustering methods yielded conflicting results but seemed to more strongly support a lack of genetic structure. BAPS, the no-admixture model in TESS, and STRUCTURE inferred K = 1 as the most likely number of clusters. In contrast, the uncorrelated frequency model in GENELAND found K = 2 and identified a north-south division (Fig 5). This north-south clustering pattern was also suggested by the CAR admixture model in TESS and less clearly by the BYM model, but in both cases using a low posterior probability threshold of 0.5, and was likewise apparent in the results of the correlated frequency model in GENELAND (K = 2 estimated in runs with K fixed to four, the value inferred in runs with varying K) but in which many individuals were assigned with probability < 0.5 (not shown). Progressive partitioning did not reveal additional differentiation within the two clusters inferred by the uncorrelated frequency model in GENELAND.

In the sPCA, one global pattern was identified in the eigenvalues barplot and the decomposition of each eigenvalue into its spatial autocorrelation and variance components (Fig D in S1 File). Similar to the result obtained with the uncorrelated frequency model in GENELAND, the first global component showed a north-south differentiation in Portugal (Fig 5).

### Genetic Variation

In the stone marten, the microsatellite loci showed low to moderate polymorphism. In the whole population, the number of alleles per locus varied from three to nine and the values of observed and expected heterozygosity ranged from 0.314 to 0.755 and from 0.349 to 0.845, respectively. The expected heterozygosity was higher than observed heterozygosity in all loci, with four of them (Mf2.13, 3.7, 8.7 and 8.10) deviating significantly from HWE (P < 0.05 after Bonferroni correction). The F_IS_ values per locus ranged from 0.010 to 0.262 (Table C in S1 File). With the exception of locus Mf2.13, MICRO-CHECKER did not detect evidence for scoring errors due to large allele dropout or stuttering, but suggested that null alleles may be present at four loci (Mf1.11, 2.13, 4.17 and 8.7). Significant linkage disequilibrium (LD) was only detected between Mf1.1 and Mf8.7 (P < 0.05 after Bonferroni correction).

The observed deviations from HWE and LE, as well as the suggestion of null alleles at some loci because MICRO-CHECKER assumes HWE, may be due to the Wahlund effect. This is supported by the analyses within subpopulations, as deviations from HWE were only found at loci Mf1.1 and Mf8.10 in the North and no LD between loci was detected in any of the subpopulations after Bonferroni correction (α = 0.05). Likewise, there was no consistent evidence across subpopulations for loci with null alleles. Genetic diversity was similar among the three subpopulations (Table C in S1 File).

No evidence for temporal genetic variation was found between the samples collected in 2008–2011 and prior to 2008 (A_R_ P = 0.691, H_O_ P = 0.932, H_E_ P = 0.209, and F_ST_ P = 0.741), and thus the observed genetic patterns should not have been influenced by temporal biases in the sampling.

The power analysis showed that the microsatellite panel and sample size provided high statistical power to detect genetic differentiation if the true F_ST_ was 0.05 (100% power) or 0.01 (99% power).

For the three subpopulations, no significant signature of a bottleneck was detected using the SMM or TPM and the mode-shift test revealed a normal L-shaped distribution. Only under the IAM, significant heterozygosity excess (P < 0.05) was detected in all subpopulations by the sign and Wilcoxon tests.

The simulation in COANCESTRY indicated the triadic likelihood estimator (TrioML, [104]) as the most appropriate relatedness estimator for the data set, since it yielded a strong correlation between true and estimated values (r = 0.79) and had the least variance for all relationship categories. Overall relatedness values were 0.07 ± 0.10 for the whole data set, 0.05 ± 0.08 for the North cluster, 0.06 ± 0.10 for the South cluster, and 0.05 ± 0.09 for the Southwest cluster. These estimates indicate a low and homogeneous relatedness across the data set.

In the red fox, microsatellite genetic variation was high, with the number of alleles per locus varying between six and 59 and observed and expected heterozygosities ranging from 0.643 to 0.944 and from 0.714 to 0.971, respectively. The expected heterozygosity was higher than observed heterozygosity in all loci, but none were out of HWE or in LD after Bonferroni correction (α = 0.05). The mean value of F_IS_ was 0.047 (Table D in S1 File). MICRO-CHECKER indicated the possible presence of null alleles at four loci (FH2189, FH2541, FH3320 and PEZ16), which were also those with the highest F_IS_ values.

Some of the loci had extremely high number of alleles (Table D in S1 File), especially when compared with other red fox studies that used the same markers [55,105], even though these studies analysed samples that may be inherently less variable: three-generation silver fox pedigrees and a local population of urban red foxes, respectively. The loci in question (FH2174, FH2189 and FH2261) all have compound and imperfect repeats and a high number of alleles can be observed in such loci [106], but the presence of multiple repetitive regions in compound microsatellites may also make them more prone to PCR-generated false alleles, particularly when the different repetitive regions are adjacent [107]. Although MICRO-CHECKER did not detect stuttering artefacts at any loci, the possible presence of undetected genotyping errors at those highly polymorphic loci should be acknowledged [108]. Therefore, we applied a MAF of 0.01 to the data and the corresponding values for the summary statistics are those on the right in each column of Table D in S1 File. The comparison for each locus of the number of alleles in the original data set and using a MAF of 0.01 shows that loci FH2174, FH2189 and especially FH2261 were the richest in very rare alleles.

We performed the analyses of genetic structure, differentiation and gene flow using a MAF of 0.01 and, considering that highly polymorphic loci may reduce measures of spatial structure [109], also without either locus FH2261 or loci FH2174, FH2189 and FH2261. The results were similar in the three cases, so all analyses reported are based on the data set including all loci with a MAF of 0.01.

The power analysis showed that the microsatellite panel and sample size provided high statistical power to detect genetic differentiation if the true F_ST_ was 0.05 (100% power) or 0.01 (100% power).

### Isolation-by-distance

In the stone marten, there was a weak but significant relationship between genetic and geographic distance for the whole data set and within the North and Southwest, while it was not consistently significant within the South for the two genetic distances used (Table 2). In the red fox, there was a weak but significant relationship between genetic and geographic distance throughout Portugal (Table 3).

**Table 2:** Results of IBD tests in the stone marten for the whole data set and for each subpopulation.

	Portugal	North	South	Southwest
GD_Rousset vs GEOD_Log: R_xy_ (P-value)	0.252 (0.000)	0.121 (0.002)	0.073 (0.080)	0.135 (0.010)
GD_Nason vs GEOD_Log: R_xy_ (P-value)	-0.217 (0.000)	-0.190 (0.000)	-0.058 (0.001)	-0.096 (0.007)

Shown are the Mantel correlation coefficient (Rxy) of the regression of Rousset’s genetic distance [82] and Nason’s kinship estimator [83] against log-transformed geographic distance, and the respective P-values

**Table 3:** Results of IBD tests in the red fox throughout Portugal using the whole data set and with a minimum allele frequency (MAF) of 0.01.

	Portugal	Portugal (MAF ≥ 0.01)
GD_Rousset vs GEOD_Log: R_xy_ (P-value)	0.074 (0.001)	0.072 (0.000)
GD_Nason vs GEOD_Log: R_xy_ (P-value)	-0.086 (0.000)	-0.085 (0.000)

Shown are the Mantel correlation coefficient (R_xy_) of the regression of Rousset’s genetic distance [82] and Nason’s kinship estimator [83] against log-transformed geographic distance, and the respective P-values

### Genetic Differentiation

Genetic differentiation between stone marten subpopulations was moderate and relatively similar across pairwise comparisons (Table 4). The lowest mean values were observed between South and Southwest and the highest were between North and Southwest. In the red fox, the estimates of genetic differentiation for the north-south discontinuity inferred by the uncorrelated frequency model in GENELAND were low (F_ST_: 0.011±0.004, G”_ST_: 0.077±0.037, D_EST_: 0.066 ±0.034; all significant, P < 0.05).

**Table 4:** Estimates of genetic differentiation between the stone marten subpopulations.

Pairwise comparison	F_ST_	G”_ST_	D_EST_
North vs South	0.053±0.020	0.131±0.052	0.083± 0.037
North vs Southwest	0.066±0.009	0.164± 0.034	0.105± 0.028
South vs Southwest	0.049±0.014	0.114± 0.036	0.069±0.026

(all significant, P < 0.05)

### Recent Migration

The BAYESASS estimates of recent migration rates between adjacent stone marten subpopulations were low (mean values of 0.02–0.03), with the exception of that for immigration from the South into the Southwest (mean value of 0.11) (Table 5; ESS > 20,000 for all migration rate estimates). In both the North and the South, the 95% HPD intervals for the proportion of non-immigrant included one. GENECLASS2 estimated two first-generation migrants in the South and one in the North. Using the ‘most likely’ criterion [110,111], 49 of the 52 individuals (94%) in the North, 47 of the 69 individuals (68%) in the South, and 27 of the 36 individuals (75%) in the Southwest, were assigned to the area in which they were sampled. The BAYESASS estimates of migration rates between the two red fox clusters identified by the uncorrelated frequency model in GENELAND were low. Posterior estimates of the proportion of immigrants from the north into the south and vice-versa were, respectively, 0.061 ± 0.028 (95% HPD interval: 0.008–0.114) and 0.038 ± 0.023 (95% HPD interval: 0.000–0.081), with ESS > 5,000 for all estimates. Only one individual, in the southern cluster, was inferred by GENECLASS2 to be a first-generation migrant. Using the ‘most likely’ criterion, GENECLASS2 supported the GENELAND assignment for 46 of the 55 individuals (84%) in the northern cluster and 75 of the 88 individuals (85%) in the southern cluster.

**Table 5:** BAYESASS estimates of the mean posterior distribution for contemporary migration rates among stone marten subpopulations.

	Into		
	North	South	Southwest
**From**			
North	-	0.033 ± 0.020 (0, 0.069)	ND
South	0.020 ± 0.016 (0, 0.051)	-	0.108 ± 0.055 (0.010, 0.211)
Southwest	ND	0.022 ± 0.016 (0, 0.052)	-

Sources are listed in the left-hand column and subpopulations receiving immigrants are listed across the second row. Values are mean ± SD and within parenthesis are the 95% HPD intervals. ND is not done, as the South area separated the North and Southwest areas.

## Discussion

In this study, we used a combination of Bayesian and multivariate techniques to characterise population structure and reveal spatial genetic patterns for further investigation in two common mesocarnivores, the stone marten and the red fox, across Portugal. We wanted to see if their genetic structure is consistent with their differences in ecological plasticity in the Iberian Peninsula, and our results are compatible with the literature reporting stricter ecological requirements of the stone marten [36,42]. We also wished to illustrate an approach to explicitly delineate the boundaries of consistently identified genetic units, based on a consensus clustering solution of different Bayesian algorithms (Fig 3). This approach allows refining the spatial partitioning of subpopulations compared with that estimated by individual methods (Fig 2). To our knowledge, the use of a consensus solution among Bayesian clustering methods to spatially delineate and characterise population structure was introduced by [35], but has not been used in subsequent studies.

Here, the consensus solution was obtained by combining the results of three powerful and popular spatially explicit Bayesian clustering programs: BAPS, GENELAND and TESS [30,35,63,64]. It has been shown that these algorithms can outperform edge detection methods, such as Monmonier’s algorithm and Wombling, at inferring genetic boundaries [64,112]. It is also recognized that different Bayesian clustering techniques should be used to investigate the spatial genetic structure in a data set, in order to evaluate the reliability and robustness of the results [31,63,113]. Spurious conclusions may be drawn when only one method is used [31]. Different clustering algorithms can produce different solutions due to differences in the underlying models and prior assumptions [63]. Moreover, comparative studies of the relative performance of Bayesian clustering models have shown that they have different strengths and weaknesses, depending on the spatial genetic patterns present and on factors such as gene flow, dispersal distance, demography and population dynamics [31,63,64]. For instance, it has been noted that GENELAND may be particularly efficient when gene flow is low and when genetic discontinuities correspond to simply shaped boundaries [28,64,112]. More generally, it is now clear that differences in performance are data set specific; for example, in a study on the genetic structure of roe deer *Capreolus capreolus* [114] GENELAND identified two clusters very weakly differentiated (F_ST_ = 0.008), while a study on reindeer *Rangifer tarandus* [35] reported the inability of GENELAND to differentiate clusters at F_ST_ = 0.02. Conversely, a common problem of Bayesian clustering models is that they may overestimate genetic structure in the presence of isolation-by-distance, especially when the IBD pattern is strong [31,64]. Although models vary in their susceptibility to this problem, they can produce consistent but incorrect clustering solutions, and thus concordance among models alone does not guarantee an accurate result [31]. Hence the importance of assessing the strength of IBD has been repeatedly emphasized [31,64]. Incorrect inferences under IBD are not only an issue for Bayesian algorithms, but also for edge detection methods [64] and for a recent multivariate clustering technique, the discriminant analysis of principal components (DAPC,[26]) [112]. Incidentally, we performed DAPC with unknown prior clusters, and the optimal number of clusters was K = 6 for the stone marten and K = 2 for the red fox (not shown). However, in both cases, the DAPC clusters were geographically meaningless and overlapped extensively, and could not be clearly related to IBD. A lack of clear geographic patterns in DAPC results has been observed in other studies [101].

When building the consensus clustering for the stone marten, we only included individuals assigned to the same cluster by no-admixture models using a threshold of 0.90 [102, 103] for the individual probabilities of cluster membership. This allowed identifying ‘core areas’ with high concentration of individuals belonging to a given genetic unit [35]. We tested less stringent cut-off probability values (0.85, 0.80), but the number of assigned individuals increased only slightly and the spatial distribution of the consensus clusters remained essentially unchanged. We did not test lower values because individuals assigning < 0.80 may be considered as potentially admixed [17,111,115]. A possibility to evaluate different thresholds and select a specific value is to use frequency-based assignment tests [95,96] and compare their assignment scores with those from the Bayesian consensus clusters [35].

To obtain a consensus pattern for further analysis, it is not required that all of the Bayesian clustering methods used yield the same optimal number of clusters K (as exemplified by the case of the stone marten). A consensus solution can be derived from the results of the methods supporting the modal K, and subsequently examined using multiple analytical tools [35]. Again, it must be emphasized that a scenario inferred by the majority of the clustering methods used in a given study is not necessarily correct, but it is a good starting hypothesis for further exploration. Here, simulations, empirically parameterized and tailored to the specific case at hand, can help to evaluate competing hypotheses and interpret empirical data [31,101,116].

### Stone marten

With the exception of the correlated frequency model in GENELAND, all of the Bayesian clustering methods divided stone martens in Portugal into three subpopulations, respectively distributed in the north, south and southwest of the country (Fig 2; Fig A in S1 File). This result is further supported by the fact that the sPCA revealed the same structuring pattern (Fig 4). However, the first global component of the sPCA only identified the north-south differentiation, while the south-southwest divide was observed in the second global component. The latter also indicated subdivision in the north, as did progressive partitioning in GENELAND (Fig B in S1 File). Accordingly, the North subpopulation showed the highest F_IS_ value (= 0.108; Table C in S1 File). Nevertheless, one of the clusters identified by the progressive partitioning in GENELAND was geographically incoherent (Fig B in S1 File), and IBD (Table 2) may have contributed to the observed substructuring. Additional local analyses with a larger sample size are warranted to address this issue.

The concurring results of the different Bayesian clustering techniques are suggestive of the presence of a marked spatial pattern, which is unlikely to be due to the weak IBD across the study area (Table 2) [31,117]. In fact, the value of Mantel’s correlation coefficient for the whole data set may be biased upwards due to the presence of population structure [118]; this idea is supported by the lower Mantel correlation within each subpopulation (Table 2). The genetic differentiation of the subpopulations was corroborated by the estimated pairwise values of F_ST_ (= 0.049–0.066) and related measures (Table 4).

The results from BAYESASS and GENECLASS2 provided additional evidence for the biological reality of the identified population structure. The BAYESASS estimates of contemporary migration rates were generally low (Table 5), suggesting that the subpopulations consist mainly of nonmigrants. The asymmetric migration between the South and the Southwest may indicate the existence of a source-sink relationship between the two subpopulations [92]. A high immigration rate from the South into the Southwest was also suggested by the results of the CAR admixture model in TESS but not by those from the BYM model and STRUCTURE (Fig A in S1 File). Therefore, it is possible that the higher estimated rate of immigration from the South into the Southwest may be due to a bias associated with the smaller sample size for the latter area. BAYESASS can overestimate mean migration rates when population sample sizes are less than 40 [92,119]. The GENECLASS2 analyses suggested low migration rates and showed no evidence of higher immigration into the Southwest. Of the three individuals identified as first-generation migrants, two in the South and one in the North, only one (in the South) had membership coefficients less than 0.8 for the area where it was sampled in both the uncorrelated frequency model in GENELAND and the no-admixture model in TESS.

The rate at which individuals are assigned to their population/region of origin by assignment tests can be used as an assessment of population genetic structure [111,120]. The relatively high proportion of individual assignments in the assignment test that was concordant with their geographic origin (68–94% per subpopulation, 78% overall) supported the consensus clustering solution. Misassignments may represent individuals with admixed ancestry or that could not be accurately assigned due to a lack of information in the data. The power of assignment tests is lower when the loci used have low to moderate genetic variation [110, 121], as was the case here for the stone marten (Table C in S1 File).

Overall, the results of the Bayesian clustering and migration analyses are consistent with long-term restricted gene flow between the subpopulations.

The bottleneck tests indicated a recent history of demographic stability for the three subpopulations, suggesting that the observed structuring is not due to increased genetic drift between bottlenecked populations or founder effects from recent range expansion [17,51]. Likewise, our results show that relatedness was not a factor influencing spatial genetic patterns.

The north-south genetic break geographically corresponds to the Tagus River (Fig 3). A north-south differentiation in Portugal was also found in two other widespread mammals in the country: the wild boar *Sus scrofa* [51] and the otter *Lutra lutra* [52]. However, in both cases the population subdivision was not strictly associated with the Tagus and other causal factors were suggested, such as recent bottlenecks [51] or historical and current fragmentation [52].

The south-southwest split was surprising, given the mobility of stone martens and the minimum distance between individuals from the two subpopulations (about six kilometres). Despite the short distance, this differentiation was detected in all Bayesian analyses and in the sPCA (Figs 2 and 5; Fig A in S1 File). The subpopulation boundary also appears to coincide with a river, the Sado River (Fig 3). A recent study of the genetic structure of the stone marten at the Iberian scale [47] identified the north-south separation mentioned above, but did not detect this break at the Sado River. This discrepancy is likely due to the fact that that study included only about half of the samples from the south of Portugal analysed here [63,122].

Rivers and other hydrologic features have been recognised as obstacles to dispersal in martens [17,23,47]. However, because the Tagus River may not currently be a significant barrier to mobile mammals [51] and the Sado River is narrower than the Tagus, future work should examine whether the existing population structure in the stone marten may reflect the history of these rivers. For instance, postglacial wet phases led to extended rivers [123] that likely restricted gene flow between populations, thereby increasing their genetic differentiation. When connectivity increased, habitat preferences [36,42] and behavioural aspects, such as site fidelity, territoriality, small home ranges, and kinship and mating bonds [124], may have prevented gene flow to completely erode historical population structure. Concurrently, the influence of the contemporary landscapes adjacent to the Tagus and Sado rivers in maintaining the observed genetic discontinuities needs to be assessed using a landscape genetic approach. Loss of preferred habitat, intensively altered landscapes and anthropogenic features may have contributed to the current population structure [16,125,126]. In this context, more samples from the extreme south of Portugal (Algarve) are needed to clarify the genetic structure in this region because population connectivity may have been affected by forest clearance for cereal cultivation at the end of the 19^th^ century and in the 1930s [127].

### Red fox

In contrast to the stone marten, here there was no clear consensus among the Bayesian clustering algorithms, but most of them (BAPS, the no-admixture model in TESS, and STRUCTURE) strongly supported the lack of population structure in Portugal. This might be expected given the high ecological plasticity of the red fox, and the adaptability and dispersal ability of the species have been invoked to explain the weak genetic structuring found in other studies [14,49].

However, the uncorrelated frequency model in GENELAND inferred the presence of a north-south division, which was also suggested by the admixture models in TESS, albeit only when using a low posterior probability threshold of 0.5, and by the sPCA (Fig 5). Discordance in results between Bayesian clustering techniques is common when F_ST_ < 0.03 [24,28]. The sPCA provides an independent inference method to check such conflicting spatial genetic patterns [63]. It is noteworthy that the north-south pattern was indicated by GENELAND, which has been singled out for its power and consistency in comparative evaluations of methods to infer genetic structure [64].

The north-south split could be an artefact due to an underlying IBD pattern, but the Mantel test showed a very weak correlation between genetic and geographic distances across Portugal (Table 3). The F_ST_ estimate for the north-south separation was low (≈ 0.01), although this may be, at least in part, due to the high heterozygosity in the data (Table D in S1 File; [128]). This hypothesis is supported by the higher values of G”_ST_ and D_EST_ (= 0.06–0.08). The BAYESASS estimates of migration rates between the two clusters identified by the uncorrelated frequency model in GENELAND were relatively low (mean values of 0.04–0.06). However, migration rate estimates from BAYESASS can be inaccurate when F_ST_ < 0.03 [119]: the posterior mean may overestimate migration rates when the true rates are low, and underestimate them when the true rates are high (> 0.1) [119]. GENECLASS2 identified a single first-generation migrant, but this result is consistent with the expected number of false positives given the specified α (= 0.01) and a total sample size of 143 individuals [96]. The individual in question had a membership probability > 0.9 in GENELAND for the area in which it was sampled. GENECLASS2 assigned 84.6% of individuals to their respective GENELAND clusters, which was higher than the percentage of stone martens assigned to their respective subpopulations (78%), despite the lower genetic differentiation among the former. This is likely explained by the fact that polymorphism of the loci in the red fox was higher (Tables C and D in S1 File) [110,121].

The north-south discontinuity is intriguing given the similar patterns observed here in the stone marten and reported previously in the otter and wild boar [51,52], even though the genetic differentiation between the two red fox clusters is lower. The lower F_ST_ could result from the high heterozygosity of the markers used, but the G”_ST_ and D_EST_ values for the subdivision in the red fox were still lower than in the stone marten. Although a large effective population size may contribute to the low genetic differentiation in the red fox, the results of the Bayesian clustering models with admixture and of the BAYESASS analysis indicate recurrent gene flow.

Thus, the north-south division may be the legacy of past landscape fragmentation associated with the flood history of the Tagus River (Fig 5) [123,129], and the pattern has been erased by increased gene flow across the divide, facilitated by the human control of the river flow rate and width, in recent times. Rivers are known to limit gene flow between red fox populations [19,49]. Alternatively or concomitantly, the genetic differentiation may be due to recent landscape fragmentation caused by the significant increase of human population density, human activities and transportation infrastructure north of the Tagus River (District of Santarém) in the last century [130]. A subsequent study, using a denser sampling from central Portugal and a landscape genetics framework, should assess the reality of the north-south split and the influence of the contemporary landscape of the region on gene flow in the red fox.

### Conclusions

The identification of hidden genetic structure is important for the management of species, even those that are currently abundant and widespread so as to guide management actions that can prevent them from becoming threatened in the future. In this study, we used a combination of Bayesian and multivariate methods to assess and compare population structure and spatial genetic patterns in two common mesocarnivores, the stone marten and the red fox, across the fragmented and heterogeneous landscape of Portugal. Our results are compatible with the known differences in ecological plasticity between the two species in the Iberian Peninsula, and are relevant for their conservation management in Portugal. Further work is needed to examine the role and influence of the Tagus and Sado rivers, as long-standing barriers with spatially and temporally variable permeability to gene flow, and of the highly modified contemporary landscapes adjacent to these rivers in shaping the observed spatial genetic patterns.

From a methodological point of view, we wanted to draw attention to an approach to delimit the spatial boundaries of consistently identified genetic units, based on a consensus clustering solution of different Bayesian algorithms. As far as we know, the explicit use of a consensus solution among Bayesian clustering methods to spatially delineate and characterise population genetic structure was introduced by [35], but has been subsequently overlooked. We suggest that it is an objective and efficient approach to obtain a conservative estimate of the spatial distribution and limits of subpopulations, and its usefulness and performance should be tested with empirical and simulated data sets. Finally, the sPCA provided a powerful complement to Bayesian clustering in the assessment of population structure and spatial genetic patterns.

## Supporting Information

S1 File Tables A-D, Figs A-D. Information on the *Martes foina* samples analysed in this study (**Table A**). Information on the *Vulpes vulpes* samples analysed in this study (**Table B**). Total and within-subpopulation microsatellite genetic diversity in Portuguese stone martens (**Table C**). Summary statistics for microsatellite loci in Portuguese red foxes (**Table D**). Cluster membership of stone marten individuals in the best run of, respectively from left to right, STRUCTURE assuming admixture and correlated allele frequencies (K = 3), TESS using the BYM admixture model (K = 3), TESS using the CAR admixture model (K = 3), and GENELAND for the correlated frequency model (K = 6) (**Fig A**). Progressive partitioning results for the northern cluster of stone martens inferred by the uncorrelated frequency model in GENELAND (**Fig B**). sPCA for stone martens (**Fig C**). sPCA for red foxes (**Fig D**).
(PDF)

S2 File Microsatellite genotypes. Microsatellite genotypes of the stone marten samples (**Table A**). Microsatellite genotypes of the red fox samples (**Table B**).
(XLS)
