# Supplementary material for: Assessing Genetic Structure in Common but Ecologically Distinct Carnivores: The Stone Marten and Red Fox
Source: PLoS One. 2016 Jan 4;11(1):e0145165. doi: 10.1371/journal.pone.0145165 (PMC4699814; doi:10.1371/journal.pone.0145165)
Supplement: S1 File — Information on the Martes foina samples analysed in this study (Table A). Information on the Vulpes vulpes samples analysed in this study (Table B). Total and within-subpopulation microsatellite genetic diversity in Portuguese stone martens (Table C). Summary statistics for microsatellite loci in Portuguese red foxes (Table D). Cluster membership of stone marten individuals in the best run of, respectively from left to right, STRUCTURE assuming admixture and correlated allele frequencies (K = 3), TESS using the BYM admixture model (K = 3), TESS using the CAR admixture model (K = 3), and GENELAND for the correlated frequency model (K = 6) (Fig A). Progressive partitioning results for the northern cluster of stone martens inferred by the uncorrelated frequency model in GENELAND (Fig B). sPCA for stone martens (Fig C). sPCA for red foxes (Fig D). (PDF) [file pone.0145165.s001.pdf]

Assessing genetic structure in common but ecologically distinct carnivores: the stone marten and red fox

Mafalda P. Basto[1,2*], Margarida Santos-Reis[1], Luciana Simões[1], Clara Grilo[3], Luís Cardoso[4], Helder Cortes[5], Michael W. Bruford[2] and Carlos Fernandes[1]

[1]Ce3C – Centre for Ecology, Evolution and Environmental Changes, Faculdade de Ciências, Universidade de Lisboa, Lisboa, Portugal; [2]Cardiff School of Biosciences, Cardiff University, Cardiff, Wales, UK; [3]Centro Brasileiro de Estudos em Ecologia de Estradas/Programa de Pós-graduação em Ecologia Aplicada, Universidade Federal de Lavras, 37200-000, Lavras, Minas Gerais, Brasil; 4Departamento de Ciências Veterinárias, Escola de Ciências Agrárias e Veterinárias, Universidade de Trás-os-Montes e Alto Douro (UTAD), Vila Real, Portugal; [5]Laboratório de Parasitologia Victor Caeiro, Instituto de Ciências Agrárias e Ambientais Mediterrânicas (ICAAM), Universidade de Évora, Évora, Portugal

*corresponding author

E-mail: mafalda.basto@gmail.com (MPB)

Supporting Information

Table A. Information on the *Martes foina* samples analysed in this study. Geographic coordinates are in WGS 84 / UTM zone 30N (EPSG: 32630). Coordinates in italics indicate approximate locations. A single asterisk indicates specimens collected before 2002; a double asterisk indicates specimens with no collection date

**No.**	**Geographic Coordinates**		**Year**	**Sex**	**Source**
	**X**	**Y**			
1	*160477*	*130844*	2002	F	CBA Tissue Bank
2	*160461*	*130833*	2002	F	CBA Tissue Bank
3	*166851*	*521873*	2002	F	CBA Tissue Bank
4	*199273*	*518410*	2002	M	CBA Tissue Bank
5	*186028*	*185342*	2002	M	CBA Tissue Bank
6	*189071*	*452519*	2002	M	CBA Tissue Bank
7	*230358*	*171816*	2002	M	CBA Tissue Bank
8	*166360*	*185276*	2002	M	CBA Tissue Bank
9	236205	148133	2002	-	CBA Tissue Bank
10	*164759*	*163149*	2002	F	CBA Tissue Bank
11	200921	105354	2003	F	CBA Tissue Bank
12	265755	322617	2003	M	CBA Tissue Bank
13	*267767*	*157423*	2003	M	CBA Tissue Bank
14	*230101*	*138094*	2003	F	CBA Tissue Bank
15	*241435*	*481498*	2003	F	Natural History Museum - Lisbon
16	159648	168045	2003	-	CBA Tissue Bank
17	213932	177848	2003	-	CBA Tissue Bank
18	*266107*	*164999*	2003	-	CBA Tissue Bank
19	*198020*	*164858*	2003	-	CBA Tissue Bank
20	165056	148159	2003	-	CBA Tissue Bank

21	267306	371200	2003	M	CBA Tissue Bank
22	165444	162776	2003	-	CBA Tissue Bank
23	151980	181582	2003	M	BRISA & Clara Grilo
24	211198	182240	2003	M	BRISA & Clara Grilo
25	161723	165882	2003	-	CBA Tissue Bank
26	227954	134578	2003	-	CBA Tissue Bank
27	167965	153012	2004	M	BRISA & Clara Grilo
28	181865	185695	2004	M	BRISA & Clara Grilo
29	216304	184861	2004	M	BRISA & Clara Grilo
30	210485	181889	2004	F	BRISA & Clara Grilo
31	198335	186407	2004	-	BRISA & Clara Grilo
32	180385	186011	2004	M	BRISA & Clara Grilo
33	221365	185071	2005	F	BRISA & Clara Grilo
34	207826	181817	2005	M	BRISA & Clara Grilo
35	186884	185619	2005	M	BRISA & Clara Grilo
36	234133	197491	2005	M	BRISA & Clara Grilo
37	164671	185161	2005	F	BRISA & Clara Grilo
38	231043	195257	2005	M	BRISA & Clara Grilo
39	196965	529461	*	M	CBA Tissue Bank
40	165655	191119	2006	-	Clara Grilo
41	*199192*	*17173*	2003	-	CBA Tissue Bank
42	*209527*	*178244*	2006	-	António Mira
43	159075	126014	2005	F	Hugo Matos
44	159043	126003	2005	F	Hugo Matos
45	240473	203335	2006	-	Clara Grilo
46	175177	186748	2007	-	Clara Grilo
47	155229	181998	2006	M	BRISA & Clara Grilo
48	*261445*	*354251*	2006	F	CBA Tissue Bank
49	268425	321418	2007	-	CERAS - Ricardo Lima
50	256275	162112	2007	-	Clara Grilo

51	197476	173690	2007	-	Fernando Ascensão
52	238469	155048	2007	-	Fernando Ascensão
53	*251116*	*274454*	2007	M	Catarina Moreira & Hélder Duarte
54	157548	178823	2007	F	BRISA & Miguel Rosalino
55	260936	206971	2007	M	BRISA & Miguel Rosalino
56	201028	185116	2007	-	BRISA & Clara Grilo & Filipe
57	*163614*	*164690*	2007	-	Fernando Ascensão
58	164431	163592	2007	-	Fernando Ascensão
59	*204736*	*161706*	2007	-	Fernando Ascensão
60	*177076*	*128103*	2007	-	Fernando Ascensão
61	*242165*	*447329*	2007	-	Sara Roque
62	*206586*	*164921*	2008	F	BRISA & Clara Grilo
63	194492	189242	2008	M	BRISA & Clara Grilo
64	*268297*	*422706*	2008	-	AFN
65	164821	167691	2008	M	BRISA & Clara Grilo
66	236993	187207	2008	F	Ana Galantinho & António Mira
67	*246220*	*327301*	2008	M	CRSE - Ricardo Brandão
68	213005	173594	2008	M	Ana Galantinho & Filipe Carvalho
69	*243373*	*446347*	2008	F	AFN - José Faustino
70	183172	567783	2008	F	Simone Lampa
71	184480	185113	2008	M	BRISA & Clara Grilo
72	174984	127281	2008	-	BRISA & Fernando Ascensão
73	229600	141545	2008	-	BRISA & Clara Grilo
74	145548	114593	2009	-	Estrela Matilde
75	155832	181689	2008	M	BRISA & Francisco Fonseca
76	141637	119626	2008	M	Cláudia Matos
77	189375	148389	2009	M	Clara Grilo
78	228391	455606	2008	-	Grupo Lobo - Duarte Cadete
79	245158	443939	2008	-	Grupo Lobo - Sara Roque
80	*259474*	*441483*	2009	M	CBA Tissue Bank

81	*179484*	*123419*	2009	F	BRISA & Clara Grilo
82	*242649*	*65198*	2010	M	Mafalda P. Basto & Miguel Rosalino
83	*247768*	*441311*	2009	-	Francisco Fonseca
84	224032	125004	2009	-	BRISA & Diana Reto
85	*180254*	*171144*	2008	-	Francisco Fonseca
86	*161235*	*202954*	2010	-	Mafalda P. Basto & Paula Gonçalves
87	259596	239632	2010	-	Ana Galantinho & Filipe Carvalho
88	232715	303563	2010	F	EP - Castelo Branco - José Martins
89	293404	342403	2010	M	EP - Castelo Branco - José Martins
90	201080	324189	2010	M	EP - Castelo Branco - José Martins
91	299296	325341	2010	-	EP - Castelo Branco - José Martins
92	236783	349137	2010	F	EP - Castelo Branco - José Martins
93	169535	185813	2009	M	BRISA & Clara Grilo
94	*188299*	*565999*	2010	-	Helena Rio Maior
95	*172634*	*115269*	2011	-	Iván Prego Alonso & Sofia Eufrásio
96	*225911*	*410749*	2010	F	CERVAS - Ricardo Brandão
97	*251278*	*386868*	2006	M	CERVAS - Ricardo Brandão
98	*252251*	*383537*	2009	M	CERVAS - Ricardo Brandão
99	*252257*	*383555*	2008	F	CERVAS - Ricardo Brandão
100	*344728*	*506998*	2008	M	CERVAS - Ricardo Brandão
101	*264578*	*378437*	2010	F	CERVAS - Ricardo Brandão
102	*259357*	*382602*	2009	M	CERVAS - Ricardo Brandão
103	*250688*	*388110*	2009	F	CERVAS - Ricardo Brandão
104	*210712*	*459269*	2010	-	EP - Aveiro - Sofia Vasques
105	*178427*	*380548*	2005	M	Carlos Fonseca - Univ. Aveiro
106	*247609*	*365250*	*	F	CERVAS - Ricardo Brandão
107	*250697*	*349416*	2004	M	CERVAS - Ricardo Brandão
108	*246224*	*373509*	2004	F	CERVAS - Ricardo Brandão
109	152672	123753	2010	M	LandGen - Luciana Simões & Fernando Ascensão
110	156456	121343	2010	M	LandGen - Luciana Simões & Fernando Ascensão

111	151740	111885	2010	F	LandGen - Luciana Simões & João Bernardo
112	151454	118483	2010	F	LandGen - Luciana Simões & João Bernardo
113	147271	85090	2010	M	LandGen - Luciana Simões & João Bernardo
114	149348	78678	2010	M	LandGen - Luciana Simões & João Bernardo
115	148452	86661	2010	M	LandGen - Luciana Simões & João Bernardo
116	156956	78197	2010	M	LandGen - Luciana Simões & João Bernardo
117	158623	85951	2010	M	LandGen - Luciana Simões & João Bernardo
118	151830	110035	2010	F	LandGen - Luciana Simões & Jacinta Mullins
119	147256	109963	2010	F	LandGen - Luciana Simões & Jacinta Mullins
120	145097	107233	2010	F	LandGen - Luciana Simões & Jacinta Mullins
121	149889	103921	2010	F	LandGen - Luciana Simões & Jacinta Mullins
122	148925	102023	2010	M	LandGen - Luciana Simões & Jacinta Mullins
123	160649	93076	2010	F	LandGen - Luciana Simões & Jacinta Mullins
124	152336	101955	2010	M	LandGen - Luciana Simões & Jacinta Mullins
125	165833	115668	2010	-	LandGen - Luciana Simões & Fabiana Marques
126	166319	120111	2010	F	LandGen - Luciana Simões & Fabiana Marques
127	167183	123586	2010	M	LandGen - Luciana Simões & Fabiana Marques
128	168838	146464	2010	F	LandGen - Luciana Simões & Fabiana Marques
129	170902	157949	2010	-	LandGen - Luciana Simões & Fabiana Marques
130	159630	107065	2011	F	LandGen - Luciana Simões & Fabiana Marques
131	158155	111725	2011	M	LandGen - Luciana Simões & Fabiana Marques
132	163845	90848	2011	F	LandGen - Luciana Simões & Fabiana Marques
133	193574	197085	2011	M	LandGen - Luciana Simões & Ana Catarina Silva
134	182245	124910	2011	M	LandGen - Luciana Simões & Fabiana Marques
135	185816	136300	2011	M	LandGen - Luciana Simões & Fabiana Marques
136	196559	161488	2011	M	LandGen - Luciana Simões & Fabiana Marques
137	196536	161530	2011	F	LandGen - Luciana Simões & Mergulho
138	190839	155513	2011	M	LandGen - Luciana Simões & Fabiana Marques
139	194285	149479	2011	M	LandGen - Luciana Simões & Fabiana Marques
140	191920	157814	2011	-	LandGen - Luciana Simões & Fabiana Marques

141	183623	168127	2011	F	LandGen - Luciana Simões & Fabiana Marques
142	198465	162968	2011	M	LandGen - Luciana Simões & Fabiana Marques
143	163201	84841	2011	F	LandGen - Luciana Simões & Jacinta Mullins
144	147871	88587	2011	F	LandGen - Luciana Simões & Jacinta Mullins
145	*202895*	*528900*	**	-	ICNF / PNPG Tissue Bank
146	*278276*	*508055*	**	-	ICNF / PNPG Tissue Bank
147	*187040*	*512015*	**	-	ICNF / PNPG Tissue Bank
148	*250308*	*438031*	**	-	ICNF / PNPG Tissue Bank
149	*185957*	*527737*	**	-	ICNF / PNPG Tissue Bank
150	*302487*	*546701*	**	-	ICNF / PNPG Tissue Bank
151	*202902*	*528871*	**	-	ICNF / PNPG Tissue Bank
152	*202912*	*528945*	**	-	ICNF / PNPG Tissue Bank
153	*202870*	*528897*	**	-	ICNF / PNPG Tissue Bank
154	*170138*	*536199*	**	-	ICNF / PNPG Tissue Bank
155	*243752*	*376376*	**	-	ICNF / PNPG Tissue Bank
156	*202896*	*528949*	**	-	ICNF / PNPG Tissue Bank
157	*294383*	*366008*	**	-	ICNF / PNPG Tissue Bank
158	244368	444466	2011	-	Sara Roque
159	238982	449181	2011	M	Sara Roque & Mónia Nakamura

Table B. Information on the *Vulpes vulpes* samples analysed in this study. Geographic coordinates are in WGS 84 / UTM zone 30N (EPSG: 32630). Coordinates in italics indicate approximate locations. A double asterisk indicates specimens with no collection date.

**No.**	**Geographic Coordinates**		**Year**	**Sex**	**Sample Source**
	**X**	**Y**			
1	197944	76806	2003	-	BRISA
2	173569	129759	2004	-	BRISA
3	260313	206609	2006	M	Clara Grilo
4	199291	186024	2006	F	BRISA & Clara Grilo
5	191976	189100	2007	F	BRISA & Clara Grilo
6	244586	205066	2007	-	BRISA & Clara Grilo
7	235895	162750	2007	-	Fernando Ascensão
8	163341	141165	2007	-	Fernando Ascensão
9	198115	123775	2007	-	Clara Grilo
10	170389	185914	2007	F	BRISA
11	297164	128997	2007	-	CBA Tissue Bank
12	155735	185727	2007	-	Clara Grilo
13	153466	373487	2008	F	AFN
14	150076	322284	2008	F	AFN
15	165405	375378	2008	M	AFN
16	170941	374686	2008	F	AFN
17	199011	19012	2008	F	AFN
18	143879	12668	2008	F	AFN
19	137668	207820	2008	F	Mafalda P. Basto, Paula Gonçalves & Iván Prego Alonso
20	150464	256847	2007	-	Paula Gonçalves

21	135579	217131	2007	-	Paula Gonçalves
22	195786	566183	2007	M	Helena Rio Maior
23	274737	212143	2008	F	Fernando Ascensão
24	286165	456756	2008	F	AFN
25	169355	22334	2008	F	AFN
26	302562	423061	2008	M	AFN
27	263671	411921	2008	M	AFN
28	183920	120739	2008	M	BRISA - Pacheco
29	224555	187060	2008	M	BRISA - Fernandes
30	*208958*	*184228*	2008	-	António Mira
31	261655	161221	2008	-	AFN
32	151455	179610	2008	M	AFN
33	157938	176253	2008	M	BRISA
34	258121	215767	2009	M	Assoc. Caçadores da Orada
35	185435	169497	2009	M	Assoc. Caçadores e Pescadores de São Cristóvão
36	232409	415915	2009	-	Clube de Caça e Pesca Os Amigos de São Miguel
37	277502	415620	2009	M	Clube de Caça e Pesca Póvoa do Concelho
38	159243	255211	2009	F	Assoc. Caçadores "Os Raposeiros" Alpiarça
39	192431	516726	2009	F	Clube de Caçadores da Póvoa do Lanhoso
40	152547	277840	2009	M	APGVN - Francisco Barros
41	113515	174981	2009	M	AFN
42	*303930*	*534371*	2009	M	Luís Cardoso
43	191177	437955	2009	M	AFN
44	163371	507680	2008	F	Luís Cardoso
45	*271776*	*130686*	2009	-	Clube de Caça e Tiro de Santo Amador
46	155952	489133	2008	F	AFN
47	175615	551091	2008	F	AFN
48	180177	540717	2008	F	AFN
49	140770	327216	2009	M	Clube de Caça e Pesca de Monte Redondo
50	214645	43871	2008	M	AFN

51	126076	314004	2009	F	Clube de Caçadores do Concelho da Marinha Grande e Clube de Caça e Pesca da Vieira
52	289871	213914	2008	M	BRISA
53	*232133*	*479133*	2008	-	Luís Cardoso
54	83806	196713	2009	-	APGVN
55	98933	222556	2009	M	AFN
56	96936	236595	2009	M	AFN
57	150729	114436	2008	F	Cláudia Matos
58	270607	161774	2009	F	Marta Cruz
59	130622	285885	2009	M	APGVN - Francisco Barros
60	*182327*	*518892*	2008	-	Luís Cardoso
61	168339	153882	**	-	BRISA
62	*258088*	*315673*	2008	M	CRSE - Ricardo Brandão
63	*336064*	*511797*	2008	F	CRSE - Ricardo Brandão
64	92171	238261	2010	M	Oestecaça
65	115772	256677	2010	F	Oestecaça
66	183914	468645	2009	M	Assoc. Caçadores do Vale do Tâmega
67	233746	360412	2010	F	Grupo Desportivo e Cultural Sobralense
68	205545	523360	2010	M	Assoc. Caça e Pesca de Ruivães
69	150841	241310	2010	M	Assoc. Caçadores de Benfica do Ribatejo
70	251709	266168	2010	-	Assoc. Caçadores e Pescadores de Alagoa
71	251388	265881	2010	-	Assoc. Caçadores e Pescadores de Alagoa
72	230072	506298	2009	M	Câmara Municipal de Ribeira de Pena
73	*305307*	*509708*	2009	F	Luís Cardoso
74	175881	500541	2010	M	Clube de Caçadores de Santa Tecla
75	213407	197554	2010	F	Clube Caçadores de Santana do Campo
76	148698	288062	2010	F	Clube de Caça e Pesca de Mira de Aire
77	237347	19110	2010	M	Clube de Caçadores Pacíficos de Santo Estevão
78	246295	331930	2010	F	Assoc. de Caça e Pesca do Vale Santo
79	161725	324103	2009	-	Clube de Caça de Sicó

80	302944	530469	2010	M	Clube de Caça Zoio
81	*150590*	*523660*	2010	M	Assoc. de Caça e Pesca de Vila Franca
82	194764	97367	2010	F	Assoc. Caçadores Livres do Concelho de Aljustrel
83	150686	295163	2010	F	Caçadores de São Mamede
84	120958	223665	2010	M	Assoc. Caçadores de Arruda dos Vinhos
85	236800	482332	2009	M	Luís Cardoso
86	173612	269560	2010	M	Assoc. Caçadores Os Mirones
87	303888	336224	2010	F	Clube de Caça e Pesca Beira Erges
88	236960	468385	2010	-	Assoc. Caçadores de Galafura & Dr Luís Cardoso
89	160749	297542	2009	F	LX-CRASPEM
90	196917	175185	2009	F	LX-CRASPEM
91	125692	244090	2009	F	LX-CRASPEM
92	116844	224566	2010	-	Assoc. Caçadores Arruda dos Vinhos
93	234354	275890	2010	F	Mafalda P. Basto & Miguel Rosalino
94	143796	389789	2009	M	Assoc. Caçadores de Mira
95	*243166*	*165522*	2008	-	Helder Cortes
96	*230624*	*187271*	2008	-	Helder Cortes
97	*204985*	*151351*	2008	-	Helder Cortes
98	*224869*	*160828*	2008	-	Helder Cortes
99	219594	301861	2010	F	Clube Pinheiro Bravo - Proença-a-Nova
100	139306	46701	2010	F	Clube Cultural e Recreativo Os Amigos da Carrapateira
101	141112	44794	2010	F	Clube de Caçadores de Quelfes
102	170224	39492	2009	M	Assoc. de Caça e Pesca de Alferce
103	213133	51951	2010	F	Assoc. de Caça Serro das Águias
104	190089	293520	2010	M	Assoc. Caçadores de Fontes
105	251450	438638	2010	M	Assoc. de Caça e Pesca de Sernancelhe
106	308538	491186	2010	M	Assoc. de Caça e Pesca de Castro Vicente, Porrais e Vilar Seco
107	332871	486869	2010	M	Assoc. Caçadores de Brunhosinho
108	271165	195064	2010	F	Clube de Caça dos Bombeiros do Alandroal
109	161403	497266	2010	M	Assoc. de Caça e Pesca de Viatodos

110	164413	408359	2010	M	Assoc. Caçadores da Branca
111	270824	373708	2010	F	Assoc. Caça e Pesca do Concelho de Belmonte
112	*353288*	*505279*	2010	-	Câmara Municipal de Miranda do Douro
113	262962	535623	2010	M	Assoc. Cultural R.D. de Santo António de Monforte & Luís Cardoso
114	166953	516566	2010	M	Assoc. das Quatro Freguesias
115	237514	400889	2010	F	Clube de Caça e Pesca de Mangualde
116	219260	115659	2009	M	Assoc. Caçadores de Terreno Livre do Distrito de Beja
117	264457	267772	2010	M	Assoc. Caça e Pesca de São Salvador da Aramenha
118	154818	201127	2010	F	Assoc. de Caçadores de Canha
119	210172	285265	2009	M	Assoc. de Caçadores do Concelho de Mação
120	248316	518733	2010	M	Assoc. Desportiva e Recreativa, Tiro, Caça e Pesca de Ribeira de Oura
121	246805	515445	2010	F	Assoc. Desportiva e Recreativa, Tiro, Caça e Pesca de Ribeira de Oura
122	355031	516012	2010	M	APATA - Mogadouro
123	181937	18773	2009	M	Os Bons Caçadores da Mesquita
124	150503	308318	2010	M	Os Bons Caçadores da Mesquita
125	151321	307782	2010	F	Assoc. de Caça e Pesca da Caranguejeira
126	237932	52440	2009	M	Assoc. de Caça e Pesca da Caranguejeira
127	266051	525070	2009	F	Clube de Caçadores Vale Largo
128	240303	268055	2009	M	Assoc. Caça Vilarandelo
129	219072	155070	2009	M	Assoc. Caçadores Pescadores do Outeiro
130	183944	279357	2010	F	Câmara Municipal de Constância
131	144250	79878	2010	M	Clube de Caçadores e Pescadores do Sudoeste Alentejano
132	202645	431633	2010	-	Grupo Lobo - Sara Roque
133	*220761*	*465454*	2009	-	Clube de Caça e Pesca de Mesão Frio & Luís Cardoso
134	*123888*	*312006*	2010	F	Clube de Caçadores do Concelho da Marinha Grande e Clube de Caça e Pesca da Vieira
135	195198	453607	2009	-	Clube de Caça e Pesca de Entre Douro e Paiva

136	258794	229459	2010	F	Ana Galantinho & Filipe Carvalho
137	264221	441094	2009	-	Grupo Lobo - Sara Pinto
138	169595	443113	2010	-	EP Aveiro
139	210897	311233	2010	F	EP Castelo Branco - Jorge Gonçalves
140	154292	130185	2008	-	BRISA & Clara Grilo
141	182377	140423	2011	-	Luciana Simões
142	241535	235323	2011	-	Mafalda P. Basto & Iván Prego Alonso
143	153925	121138	2011	-	Luciana Simões

Table C. **Total and within-subpopulation microsatellite genetic diversity in Portuguese stone martens** (n=157 individuals), as measured by the number of alleles per locus (N_A), observed heterozygosity (H_O), unbiased expected heterozygosity (UH_E), inbreeding coefficient (F_{IS}), allelic richness (A_R) and private allelic richness (pA_R). Significant F_{IS} values ($P < 0.05$) are indicated by an asterisk

**Locus**	**N_A**	**H_O**	**UH_E**	**F_{is}**
**Mf3.7**	4	0.312	0.349	0.113*
**Mf8.7**	4	0.599	0.720	0.172*
**Mf8.8**	9	0.713	0.783	0.099*
**Mf3.2**	5	0.631	0.674	0.059
**Mf8.10**	7	0.637	0.718	0.107*
**Mf4.17**	9	0.752	0.844	0.107*
**Mf1.1**	4	0.344	0.349	0.010
**Mf6.5**	5	0.592	0.640	0.079*
**Mf4.10**	6	0.675	0.742	0.084*
**Mf1.11**	3	0.350	0.439	0.207*
**Mf1.3**	6	0.567	0.619	0.090*
**Mf2.13**	4	0.350	0.463	0.262*
**Mean**	5.5	0.544	0.612	0.116*

**Area**	**A_R**	**pA_R**	**H_O**	**UH_E**	**F_{is}**
**North (52)**	4.94	0.66	0.556± 0.049	0.622±0.043	0.108*±0.044
**South (69)**	4.39	0.06	0.534± 0.057	0.573±0.053	0.068±0.025
**Southwest (36)**	4.25	0.11	0.544± 0.050	0.573±0.048	0.052±0.037

Table D. **Summary statistics for microsatellite loci in Portuguese red foxes**: number of alleles per locus (N_A), observed heterozygosity (H_O), unbiased expected heterozygosity (UH_E), and inbreeding coefficient (F_{IS}). Original values (left) and values using an allele frequency threshold of 0.01 (right)

Locus	N_A	H_O	UH_E	F_{IS}
**FH2261**	59/39	0.944/0.930	0.971/0.969	0.031/0.041
**FH2174**	33/23	0.930/0.923	0.944/0.941	0.018/0.020
**PEZ16**	24/15	0.846/0.847	0.902/0.896	0.066/0.054
**FH2541**	20/16	0.853/0.847	0.909/0.908	0.064/0.068
**FH2302**	22/18	0.888/0.883	0.920/0.919	0.038/0.040
**FH2189**	37/24	0.881/0.864	0.935/0.927	0.061/0.068
**FH2142**	12/10	0.776/0.773	0.812/0.811	0.047/0.047
**FH3320**	12/8	0.643/0.630	0.734/0.726	0.127/0.132
**FH2613**	10/8	0.755/0.752	0.738/0.738	- 0.019/-0.019
**FH2318**	6/5	0.685/0.683	0.714/0.715	0.043/0.044
**Mean**	23.5/15.6	0.820/0.813	0.858/0.855	0.047/0.049

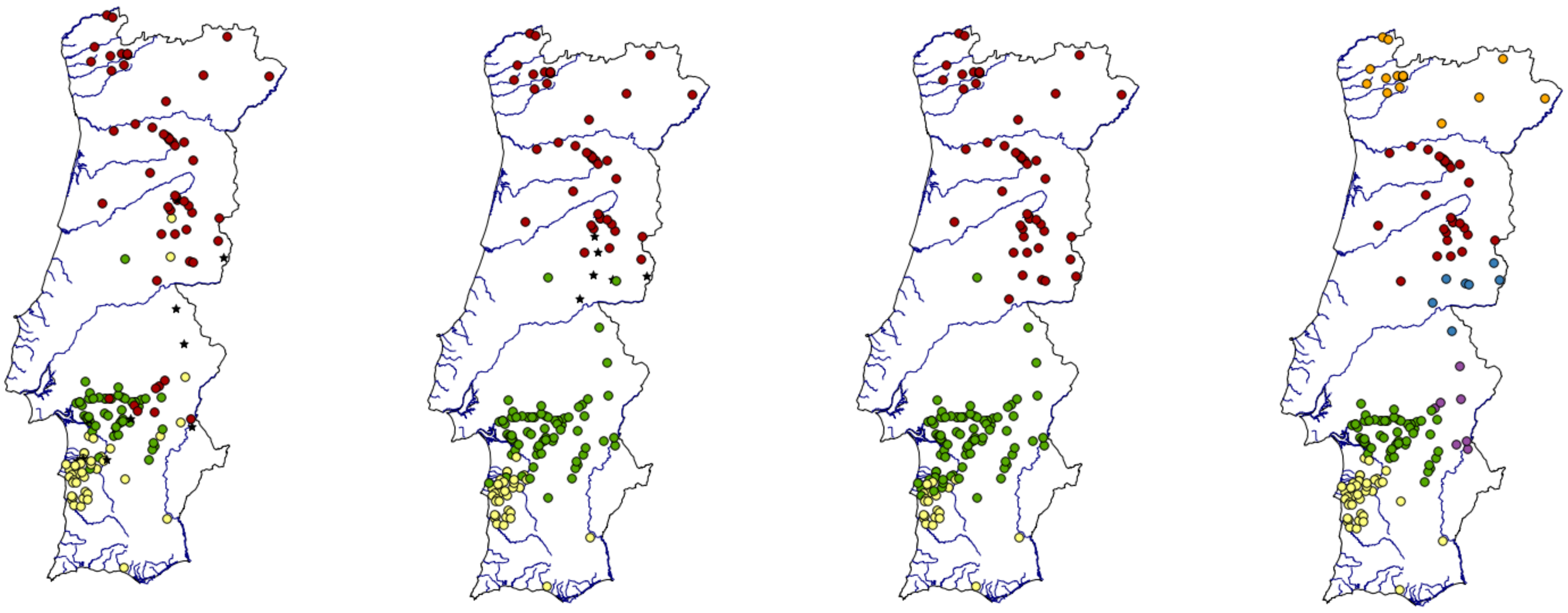

Figure A. Cluster membership of stone marten individuals in the best run of, respectively from left to right, STRUCTURE assuming admixture and correlated allele frequencies (K=3), TESS using the BYM admixture model (K=3), TESS using the CAR admixture model (K=3), and GENELAND for the correlated frequency model (K=6). The assignment threshold was 0.5, except for GENELAND in which no threshold was applied and individuals were assigned to the cluster where their ancestry proportion was highest. Stars represent non-assigned individuals (i.e., with posterior probabilities < 0.5). Lines represent country borders and main watercourses.

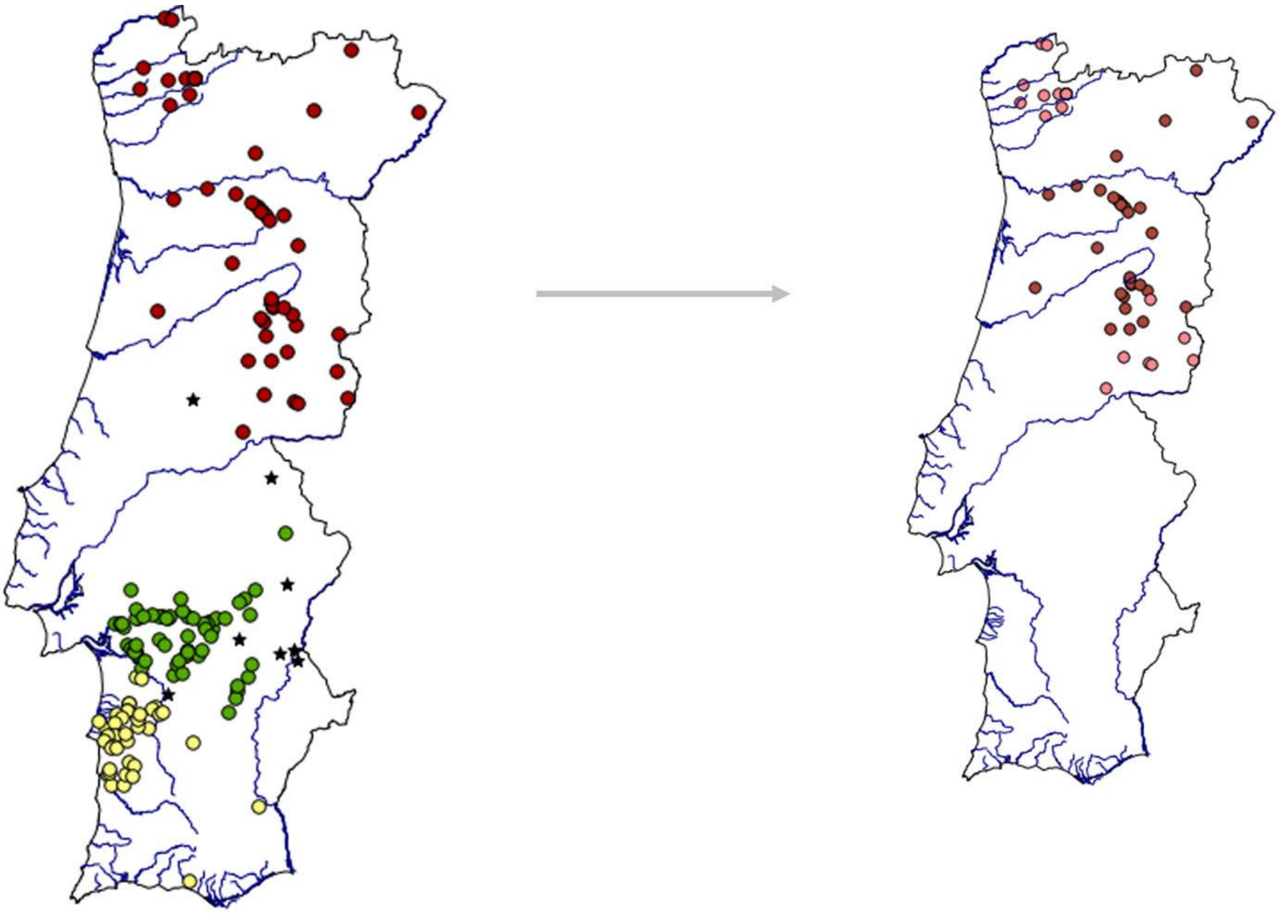

Figure B. Progressive partitioning results for the northern cluster of stone martens inferred by the uncorrelated frequency model in GENELAND. On the left, cluster membership from GENELAND K=3; on the right, northern cluster split into two additional clusters. Lines represent country borders and main watercourses.

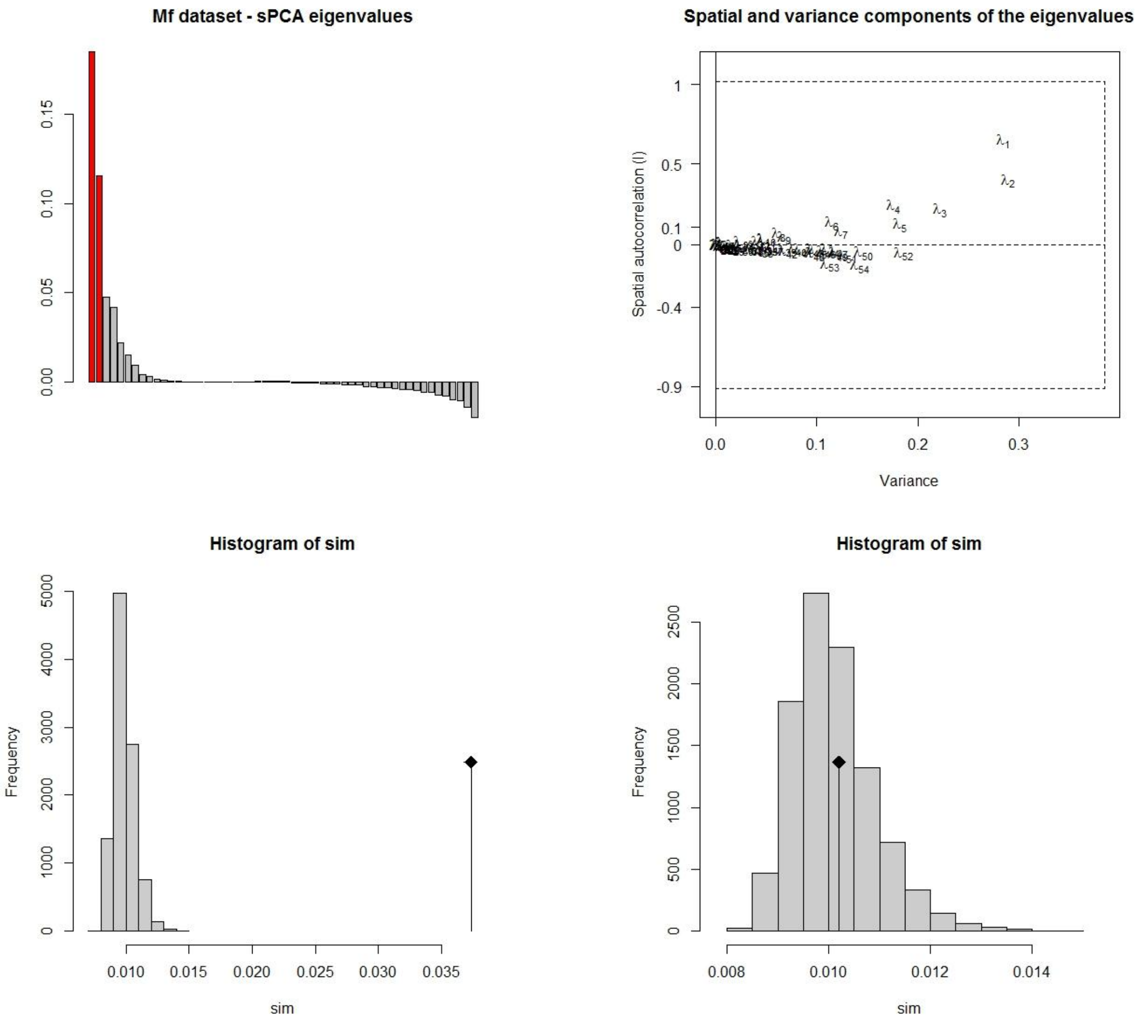

Figure C. sPCA for stone martens. Top left: the eigenvalues barplot. Positive eigenvalues (on the left) indicate global structures and negative eigenvalues (on the right) indicate local patterns. The first two positive eigenvalues (in red) are associated to the principal components that were retained; top right: decomposition of each eigenvalue into its spatial autocorrelation and variance components. Each λi (with i = 1, … , r) represents eigenvalues of sPCA, where $\lambda 1$ is the highest positive and λr is the highest negative, according to their variance and Moran's I components; bottom left: G test histogram; bottom right: L test histogram. The last two are histograms of permuted test statistics where the observed statistics is indicated by a black diamond. The G test was significant, meaning presence of spatial structure, and the L test was not significant.

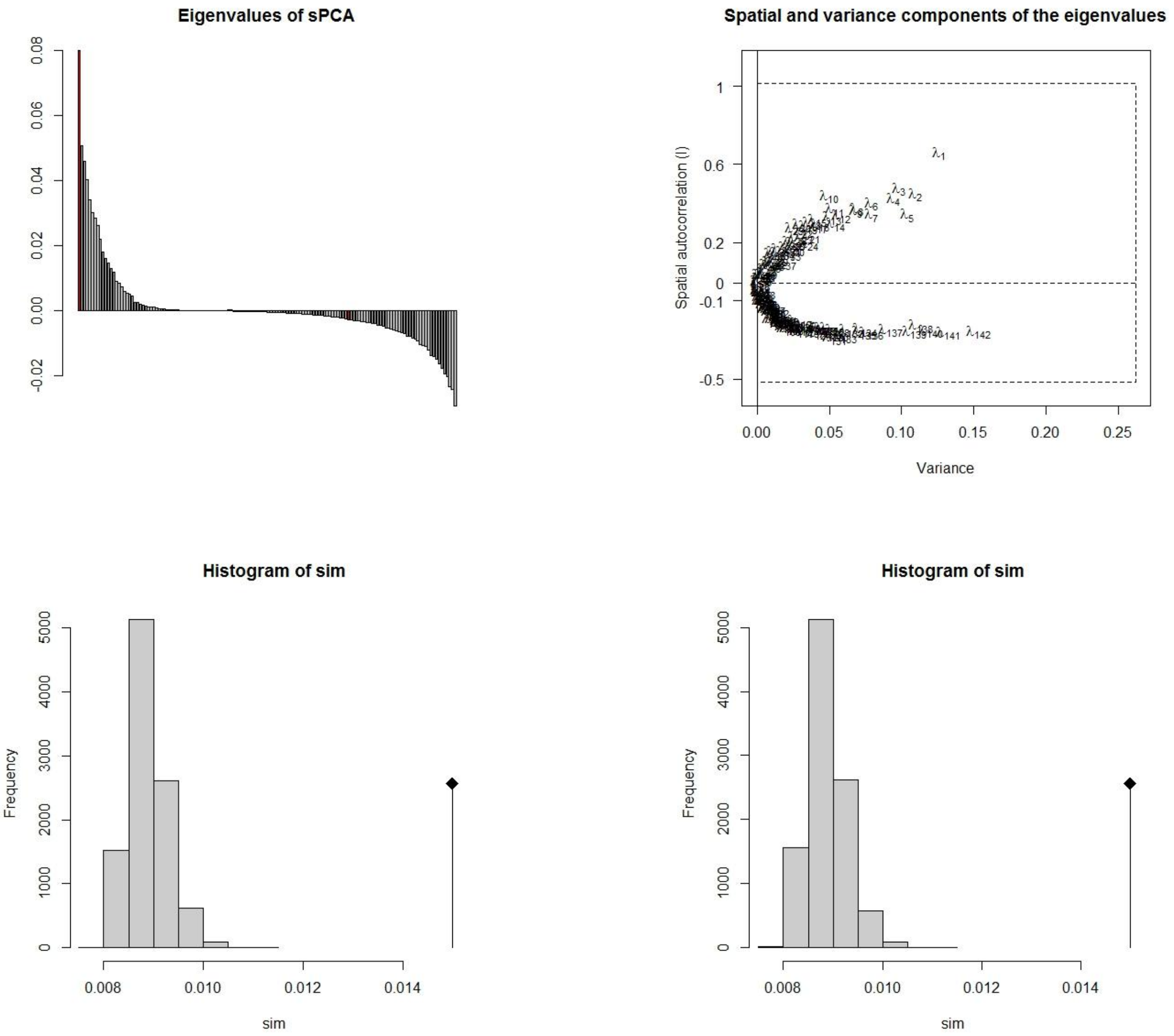

Figure D. sPCA for red foxes. Top left: the eigenvalues barplot. Positive eigenvalues (on the left) indicate global structures and negative eigenvalues (on the right) indicate local patterns. The first positive eigenvalue (in red) is associated to the principal component that was retained; top right: decomposition of each eigenvalue into its spatial autocorrelation and variance components. Each λi (with $i = 1, \dots, r$) represents eigenvalues of sPCA, where $\lambda 1$ is the highest positive and λr is the highest negative, according to their variance and Moran's I components; bottom left: G test histogram; bottom right: L test histogram. The last two are histograms of permuted test statistics where the observed statistics is indicated by a black diamond. Both tests were significant.
